# Developing Versatile Contactors for Direct Air Capture of CO_2_ through Amine Grafting onto Alumina Pellets and Alumina Wash-Coated Monoliths

**DOI:** 10.1021/acs.iecr.3c01265

**Published:** 2023-08-15

**Authors:** Quirin Grossmann, Valentina Stampi-Bombelli, Alexander Yakimov, Scott Docherty, Christophe Copéret, Marco Mazzotti

**Affiliations:** † Institute of Energy and Process Engineering, Sonneggstrasse 3, ETH Zurich, 8092 Zurich, Switzerland; ‡ Department of Chemistry and Applied Biosciences, Vladimir Prelog Weg 2, ETH Zurich, 8093 Zurich, Switzerland

## Abstract

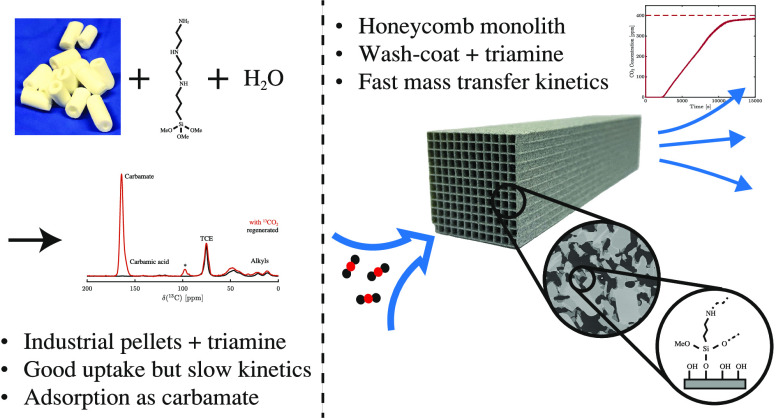

The optimization of the air–solid contactor is critical to improve the efficiency of the direct air capture (DAC) process. To enable comparison of contactors and therefore a step toward optimization, two contactors are prepared in the form of pellets and wash-coated honeycomb monoliths. The desired amine functionalities are successfully incorporated onto these industrially relevant pellets by means of a procedure developed for powders, providing materials with a CO_2_ uptake not influenced by the morphology and the structure of the materials according to the sorption measurements. Furthermore, the amine functionalities are incorporated onto alumina wash-coated monoliths that provide a similar CO_2_ uptake compared to the pellets. Using breakthrough measurements, dry CO_2_ uptakes of 0.44 and 0.4 mmol g_sorbent_^–1^ are measured for pellets and for a monolith, respectively. NMR and IR studies of CO_2_ uptake show that the CO_2_ adsorbs mainly in the form of ammonium carbamate. Both contactors are characterized by estimated Toth isotherm parameters and linear driving force (LDF) coefficients to enable an initial comparison and provide information for further studies of the two contactors. LDF coefficients of 1.5 × 10^–4^ and of 1.2 × 10^–3^ s^–1^ are estimated for the pellets and for a monolith, respectively. In comparison to the pellets, the monolith therefore exhibits particularly promising results in terms of adsorption kinetics due to its hierarchical pore structure. This is reflected in the productivity of the adsorption step of 6.48 mol m^–3^ h^–1^ for the pellets compared to 7.56 mol m^–3^ h^–1^ for the monolith at a pressure drop approximately 1 order of magnitude lower, making the monoliths prime candidates to enhance the efficiency of DAC processes.

## Introduction

Climate change is one of humankind’s greatest challenges of the 21st century, with consequences including further loss of biodiversity and strain on water resources and food production, consequently leading to mass migration, ultimately threatening each sovereign nation’s national security.^1,2^ Anthropogenic global warming is caused by rapidly increasing emissions of greenhouse gases into the earth’s atmosphere through the burning of fossil fuels.^3,4^ Stabilizing global mean temperatures to reduce these potentially devastating consequences requires global greenhouse gas emissions to reach net zero as quickly as possible. By far, the most significant of these greenhouse gases is CO_2_. Transitioning to clean technologies not dependent on fossil fuels will inevitably take time, during which technologies that reduce CO_2_ emissions immediately will be necessary.^4^ Capture from stationary, more concentrated point sources, such as power plants and industrial plants, involves mature technologies that are already being implemented with measurable success. On the other hand, carbon dioxide removal (CDR) technologies that capture CO_2_ from spatially distributed emissions, such as those from agriculture and transport, still face technical challenges. One challenge all these technologies must face is the low concentration of CO_2_ in the air, which makes it thermodynamically less favorable compared to capture from stationary sources.^5^ Nonetheless, CDR is essential in reaching net-zero goals and technologies such as direct air capture (DAC) are currently being developed both in academia and in the industry to achieve this. Current DAC processes present a promising method to achieve CDR compared to those capturing CO_2_ through plant-based materials (bioenergy with carbon capture and storage, BECCS) as land use is dramatically reduced, there is no potential conflict with food production,^6^ and unintentional emissions due to land use change are avoided.^7^ However, these advantages currently come at the cost of both higher energy demand and higher cost, which can be decreased by progressing quickly along the learning curve.^8−10^

Adsorption-based temperature-swing adsorption (TSA) processes are among the most promising DAC technologies currently in use.^11,12^ At the heart of a TSA process is the air–solid contactor, through which the air flows and where the CO_2_ is extracted. Contactors can be characterized by the type of sorbent and support material, the geometric structure thereof, and the heat integration mechanism. The choice of these characteristics can both affect and be a consequence of the chosen process, which in turn is determined by external factors such as heat and electricity availability and environmental conditions.^13^ The choice of contactor then significantly impacts the effectiveness of the process.^14^ In general, effective DAC contactors should fulfill the following criteria:

- low pressure drop
- high packing density (low spatial footprint)
- long lifetime^9^
- high affinity to CO_2_^14^
- fast heat and mass transfer.^14^

In particular, fast mass transfer was found to be of significantly higher importance than CO_2_ uptake for improving the efficiency of the DAC process.^14^

Sorbent design for DAC has received much attention in the open literature, and remarkable results have been achieved with respect to CO_2_ capacity.^15−19^ Notably, mass transfer kinetics are often overlooked or only passingly studied despite their importance for DAC. Among the most studied sorbents are amine-functionalized high-surface area oxides, which are promising materials due to a combination a high selectivity toward CO_2_ at atmospheric conditions, tolerance to humidity, and low temperature regeneration. Silica is the most common oxide used in studies of DAC sorbents due to its favorable properties in this regard. Alumina has received less attention despite its higher hydrothermal stability compared to silicas, which have been reported to lose CO_2_ capacity when exposed to steam due to collapsing of the pore structure.^20^ As steam regeneration is viewed as a viable option for DAC,^12^ this property of alumina is favorable. The γ phase of alumina also exhibits high surface area and pore volume, both of which make it an interesting candidate for adsorption applications with or without further functionalization.^18,21^ Furthermore, the environmental impact of alumina has been found to be low compared to other support materials used for DAC adsorbents.^22^

**Figure 1:**
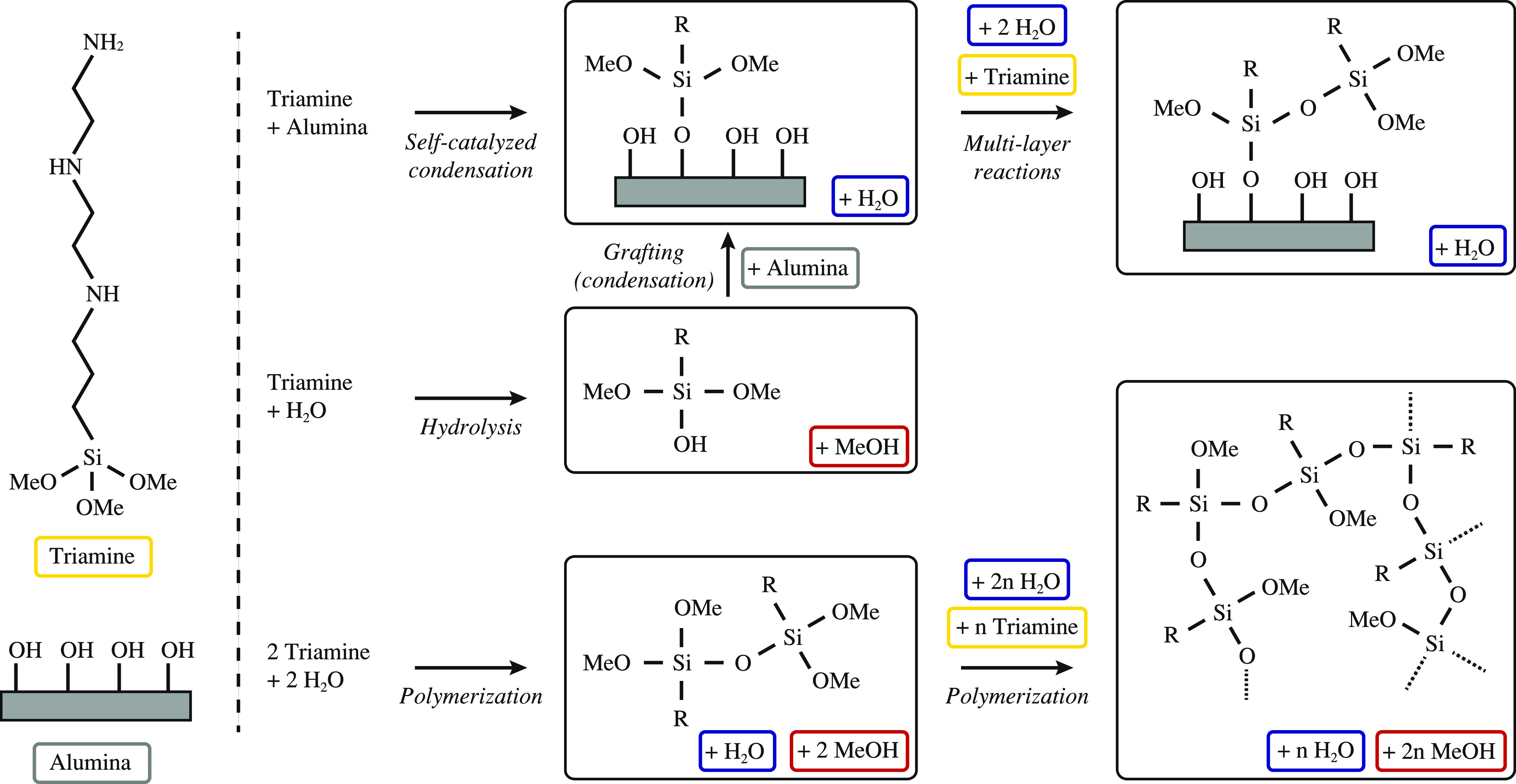
Grafting and polymerization mechanisms of triamine on alumina.

Notable work has been performed to understand the mechanisms of functionalization, adsorption, and degradation of these materials, and several extensive reviews can be found in the academic literature.^20,23−25^ The amine functionalities are mostly immobilized on support materials by either impregnation (class I) or grafting (class II).^23,26^ Impregnation involves filling a large fraction of the pore volume of the support material with amine-containing molecules, which then form hydrogen bonds with the hydroxy groups present on the surface of the support material but do not form covalent bonds. These materials generally offer high CO_2_ capacities, but the thick amine layer leads to high mass transfer resistances.^17,19,27^ Grafting involves covalently bonding amine functionalities to the support material surface by silylation with the surface hydroxy groups, typically using a molecule with methoxy or ethoxy silane groups.^28^ Typically, the wet grafting procedure is used, in which a controlled amount of water is added to the support material before grafting.^29,30^ Compared to dry grafting, higher amine loadings and therefore higher CO_2_ uptakes can be achieved.^30−35^ An overview of the functionalization mechanisms is shown in Figure 1. It is shown that water not only acts as a catalyst for the condensation reaction on the surface hydroxy groups, but it also enables polymerization.^35^ While the polymerization reaction can increase amine loading if it occurs in the context of a multilayer reaction, excessive polymerization may lead to pore blockage and thereby decrease the accessibility of the amine adsorption sites.^31^ Furthermore, excessive amounts of water in the solvent may lead to polymerization outside the support material, decreasing the grafting efficiency.^30^ Reference values have been suggested to provide optimal grafting, e.g., 1.15 monolayers^30^ or 2.5 monolayers,^32^ although the amount of water is likely specific to the support material’s pore and surface properties and to the functional group.^36^ In general, the class II materials discussed offer lower CO_2_ capacities but faster mass transfer due to reduced pore blockage.^26^ Based on findings showing that mass transfer kinetics are more important than CO_2_ capacity,^14^ this functionalization method was chosen for this work.

CO_2_ adsorption on amine-functionalized materials under dry conditions can occur through the formation of ammonium carbamate and carbamic acid. The latter can be a precursor to the formation of ammonium carbamate or stabilized via hydrogen bonds with either a neighboring carbamic acid or surface hydroxy groups.^37−43^ The presence of water enables additional species to be formed, namely ammonium bicarbonate and water-stabilized carbamic acid.^43−46^ The prevalence of these species has been found to be determined by various factors such as amine density: at high densities, ammonium carbamate tends to form, whereas at low densities, stabilized carbamic acid is prevalent.^41−43^ The formation of bicarbonate, while increasing the theoretical capacity of the sorbent through more efficient use of amines, is very slow and so may not be relevant for practical DAC processes.^46^

Most research on these materials has been performed on powders. While they are more practical to produce and to analyze in this form, they must be formed into geometries that can create efficient contactors to be useful in industrial applications. Various forms have been proposed for use in DAC, such as manifolds of pellet beds,^47^ radial flow adsorbers,^48^ honeycomb monolith structures,^49^ and fiber sorbents.^50^ Both the manifolds and radial flow adsorbers rely on pellets as an adsorbent. While these are generally regarded as the industry standard, pellets of a size relevant to the industry suffer from slow mass transfer kinetics. In contrast, honeycomb monoliths offer an alternative that offers fast mass transfer kinetics while maintaining the low pressure drops necessary for DAC. Fiber sorbents exhibit similarly promising characteristics but are a less mature technology compared to monoliths for use in DAC.^50^

For the production of pellets, pelletization of functionalized powders can be used, but it has been shown to both increase mass transfer resistance and decrease adsorption capacity.^51,52^ Additionally, the resulting pellets cannot be calcined without damaging the amine functionalities, which likely decreases the mechanical strength and therefore the lifetime of the sorbent.^53,54^ Honeycomb monoliths with amine functional groups have been successfully 3D-printed, although using grafted powders in the paste did not yield successful printing, thus implying that the monoliths had to be post-functionalized.^55^ This technique has also been successfully used on other monoliths, with the advantage of producing stable honeycombs at the expense of a more complex functionalization procedure.^49,56^

This work focuses on producing mechanically stable, industrially relevant DAC sorbents using methods developed in the open literature and industry. Alumina is chosen as a support material due to the aforementioned hydrothermal stability. Amine functionalities are incorporated onto the alumina via grafting (class II materials) to minimize the mass transfer resistance. Two geometries of sorbents are produced and studied in the context of optimizing contactor design for DAC. Pellets represent the standard geometry of adsorbents and can therefore offer a baseline for comparison. Honeycomb monoliths promise to improve on them due to the aforementioned enhanced mass transfer kinetics and low pressure drops. To form a basis of comparison of the two contactors, process-relevant characteristics are estimated, such as isotherms and mass transfer kinetics. The results indicate that the honeycomb monoliths are indeed promising for use as DAC contactors.

## Materials and Methods

### Materials

Toluene puriss ACS reagent grade (99.8%) was purchased from Chemie Brunschwig. Anhydrous toluene (99.8%), 3-[2-(2-aminoethylamino)-ethylamino]-propyltrimethoxysilane (triamine), octyltrimethoxysilane (OTMS), and zeolite 3A were purchased from Sigma Aldrich. The reagents were stored under a N_2_ atmosphere once opened. Hollow cylindrical alumina pellets SA6578 with a constant diameter of 3 mm and nominal length of approximately 5 mm (with a range of ca. 4–7 mm) were kindly provided by Saint Gobain (France) and will henceforth be referred to as SA. The alumina wash-coat (Pural TH 200) was kindly contributed by Sasol (Germany), and the wash-coated mullite monoliths were kindly contributed by HUG Engineering (Switzerland), which together will henceforth be referred to as MAl. The monoliths were supplied in a dimension of 30 mm × 30 mm × 130 mm, with a CPSI of 100, channel size of 2 mm × 2 mm, and a wall thickness of ca. 0.4 mm. All non-labeled gases were purchased from Pangas, ^13^CO_2_ was purchased from CortecNet, 99.27% ^13^C.

### Wash Coating of Monoliths

The wash coating of the monoliths was performed by Hug Engineering, where the macroporous mullite monoliths were wash-coated using Sasol Pural TH 200 dispersed in water. The monoliths were then dipped into the dispersion, left to dry, and calcined at 500 °C for 1 h. Some measurements were performed on pure wash-coat material, i.e., not deposited on a monolith. In this case, the wash-coat material was calcined in the same manner in a beaker and will be referred to as the wash-coat material.

### Functionalization of the Support

#### Small Scale

The recipe for grafting the alumina pellets was based on that used earlier by Harlick and Sayari.^30^ The pellets were first dried for 4 h at 120 °C and 125 mbar. Four 20 mL vials were fitted with a metal mesh to separate the alumina rings from the stir bar as seen in Figure S1. Then, 1 g of dried alumina pellets was added per vial, followed immediately by 15 mL of anhydrous toluene. After 15 min of stirring in a reaction block on a hot plate at room temperature (see Figure S1), a specified amount of Micropur water was added to each vial. This was allowed to equilibrate for 1 h, after which the temperature was raised to 85 °C. Once the temperature had stabilized, a specified amount of triamine was added dropwise to each vial. This was then left for 12 h and then allowed to cool to room temperature before washing the pellets with toluene, ethanol, and diethyl ether. The functionalized pellets were then allowed to dry in ambient air for at least 24 h and stored in vials labeled SA-TRI-*x*-*y*, where *x* is the amount of triamine added in μL and *y* is the amount of water added in μL. The sample functionalized with an octyl chain was prepared in the same manner, replacing triamine with octyltrimethoxysilane (OTMS).

#### Bench-Scale Pellet Functionalization

The scaled up functionalization of the pellets was performed in a 7 L reactor. The temperature was controlled with a heat jacket connected to a thermostat. A glass stirrer was used to ensure good mixing. The whole reactor was sealed and fitted with a glass bubbler filled with silicon oil to avoid overpressure, and an argon inlet was attached to provide inert dry gas. Reactants were introduced through a septa in the lid and a syringe pump. A schematic of the setup is provided in Figure S2. A mesh was fitted to prevent the pellets from accumulating at the bottom of the reactor as seen in Figure S2. In the first step, 300 g of zeolite 3A was dried for 24 h at 150 °C and 0.125 bar. These were then added to 4 L of toluene in the reactor, which was then sealed, purged with argon, and then left for another 24 h to ensure that the toluene was dry.^57^ Simultaneously, the support material was dried as described in the small-scale procedure. The zeolites were then removed from the reactor before adding the support material to the reactor. The reactor was then purged again with Ar and the support material was left for 1 h while being stirred. A specified amount of water was then added dropwise, and the system was allowed to equilibrate for 2 h before increasing the temperature to 85 °C. The specified amount of triamine was then added dropwise to the reactor and allowed to equilibrate for 12 h. The thermostat was then set to room temperature and a small argon flow was introduced to ensure no underpressure ensued. The functionalized material was then washed with toluene, ethanol, and diethyl ether and then allowed to dry in air for 24 h.

#### Bench-Scale Monolith Functionalization

The monolith functionalization proceeded much as the pellet functionalization, albeit in a 0.5 L reactor. The hydration procedure differed in that the monoliths were first dried, then added to hot water (60–70 °C) for 2 h before being allowed to drip-dry, and then placed in a vacuum oven at 60 °C and 50 mbar until the mass increase compared to the dried sample was approximately equivalent to 1.5 monolayers of water. Meanwhile, the reactor was prepared with a mixture of toluene and triamine under anhydrous conditions. The temperature of the reactor was then increased to 85 °C before removing the stirrer and adding the monolith. The rest of the procedure was identical to that adopted for the pellets.

### Material Characterization

#### XRD

Powder XRD measurements were performed with a D2 Phaser 2nd-generation portable desktop instrument by BRUKER. For this, the pellets were crushed to a powder with a pestle and mortar. For the wash-coat material, a portion of the dispersion was placed in a beaker, allowed to dry, and calcined at 500 °C. The resulting powder was then used for the XRD measurement.

#### N_2_ Physisorption

N_2_ physisorption measurements were performed using a Microtrac Belsorp Max volumetric adsorption instrument at 77 K. The samples were pretreated at 100 °C for 3 h in vacuum after having performed CO_2_ adsorption measurements. The specific surface area was calculated using Brunauer–Emmet–Teller (BET) theory from isotherm data in the relative pressure range of 0.05–0.30. The pore volume was estimated as the single-point amount adsorbed at *P*/*P*_0_ = 0.99. Lastly, the pore size of the support material was estimated from the desorption branch of the isotherm, using the Barrett–Joyner–Halenda (BJH) method.

#### Elemental Analysis

C, H, and N elemental analysis was conducted to determine the amount of functional groups on the adsorbents. The materials were ground to a powder to enable the analysis. The measurements were performed by the Molecular and Biomolecular Analysis Service (MoBiAS) at ETH Zurich using a LECO TruSpec Micro instrument, using an average of two measurements.

#### CO_2_ Adsorption Equilibrium

Approximately 300 mg of pellets or 1 g of monolith was first pretreated at 100 °C under vacuum for 3 h and then used to measure the CO_2_ adsorption capacity in a Microtrac Belsorp Max volumetric adsorption measurement device. The adsorbed amount of CO_2_ could be determined with a relative error of less than 1%. For cases in which there was no equilibrium point at 40 Pa (equal to 400 ppm partial pressure), the adsorption was estimated by interpolation between measurement points using the modified Akima method.

#### CO_2_ Adsorption Kinetics

Approximately 100 mg of pellets was pretreated as in the equilibrium measurements and then measured using the Microtrac Belmaster adsorption rate analysis program. Inert stainless-steel beads were added to the measurement cell to increase the thermal mass and ensure isothermal conditions. Steps of 1 cm_STP_^3^ g^–1^ were used to ensure the linear isotherm assumption used by software. The measurement results were then fitted to the batch linear driving force (LDF) model to get the corresponding LDF mass transfer coefficient.

#### DRIFT Spectroscopy

Samples were ground to a powder before a small amount was placed in a heated, flow-through DRIFT cell on a bed of KBr. This cell was then placed in a Bruker Vertex 70V. Prior to a measurement, the sample was purged with N_2_ at a flow rate of 10 mL/min and heated to 120 °C for 1 h to desorb any water and CO_2_. 100 scans were then performed in a range of 4000–700 cm^–1^. For measurements of CO_2_ adsorption, the cell was first cooled to 25 °C; then, the gas was switched to 399 ppm CO_2_ in N_2_, and measurements were performed at intervals of approximately 5–10 seconds.

#### CO_2_ Adsorption for FTIR and NMR Measurements

Approximately 250 mg of powdered sample was first evacuated on a high-vac line at 10^–4^ mbar and 100 °C for 12 h before being transferred to an Ar glovebox (O_2_ < 0.5 ppm, H_2_O < 0.5 ppm). For the adsorption of CO_2_, approx. 200 mg of sample was then transferred to a 50 mL Rotaflo flask (1). Another 50 mL Rotaflo flask was filled to 1 bar with CO_2_ and attached together with a T-junction and (1) to a high-vac line. The volume of the T-junction was measured using a pressure gauge; then, a calculated amount of CO_2_ was dosed into (1) to result in a target equilibrium pressure of 0.4 mbar and left to equilibrate for at least 5 h. Subsequently, (1) was evacuated shortly and transferred back to the Ar glovebox for further preparation. The procedure was used for both ^12^CO_2_ and ^13^CO_2_ adsorption.

#### FTIR Transmittance Spectroscopy

The FTIR transmittance measurements were performed in an Ar glovebox on a Bruker Alpha FTIR spectrometer in a range of 4000–700 cm^–1^ with 16 scans. Approximately 10 mg of the previously prepared sample (with or without CO_2_ adsorption) were pressed into a pellet to enable the measurement.

#### Solid-State NMR Spectroscopy

Dynamic nuclear polarization (DNP) surface-enhanced ^13^C and ^15^N NMR measurements^58−61^ were conducted at 600 MHz (14.1 T) with a Bruker Ascend NMR spectrometer by using a 3.2 mm HX probe. For the measurements, the samples were formulated by impregnating ca. 20 mg of solid with 20 μL of TEKPol radical 16 mM solution in 1,1,2,2-tetrachloroethane (TCE), which was preliminary dried over CaH_2_, vacuum-transferred from CaH_2_, and subsequently degassed via three freeze–pump–thaw cycles prior to being introduced to the glovebox. The formulated samples were then packed in sapphire rotors for optimal microwave penetration. The measurements were conducted at 111 K by spinning the rotor at a rate of 10 kHz in a cold N_2_ flow. The ^1^H-^13^C CPMAS pulse sequence was used for the measurements. Gyrotron-generated microwaves with a power of approximately 6 W at 395 GHz were used to drive the DNP cross effect. The chemical shift was calibrated by assigning the ^13^C NMR signal of TCE (solvent) to 75 ppm.

#### Breakthrough Measurements

The breakthrough measurements were performed on the setup described previously, modified to include infrared CO_2_ sensors (Vaisala, 0–2000 ppm and 0–20%) in place of the mass spectrometer.^62^ The column used for the pellets was a 325 mm-long cylindrical column with a circular cross-section of 33 mm diameter. The column used for the monolith was 150 mm long with a square cross-section of 32 mm × 32 mm. Fleece strips were fitted around the monolith to prevent flow bypass. The pellet column was wrapped with electric heating wires, while the monolith column was fitted with a heat jacket connected to a thermostat to enable a temperature swing. Adsorption was performed with a dry feed of 392.0 ppm CO_2_ in N_2_ at 0.002 mol s^–1^ for the pellet column and at 400.1 ppm CO_2_ in N_2_ at 0.001 mol s^–1^ for the monolith. The regeneration was performed with a N_2_ purge at 100 °C for 3 h for the monolith and for 4 h for the pellets, after which no CO_2_ was observed at the outlet. To measure the desorption, the CO_2_ concentration was monitored using the 0–2000 ppm sensor for the monolith and the 0–20% sensor for the pellets at a constant molar flow rate of N_2_. The back pressure regulator was removed from the system, as the column was operated at ambient pressure.

## Results and Discussion

### Support Material

**Figure 2:**
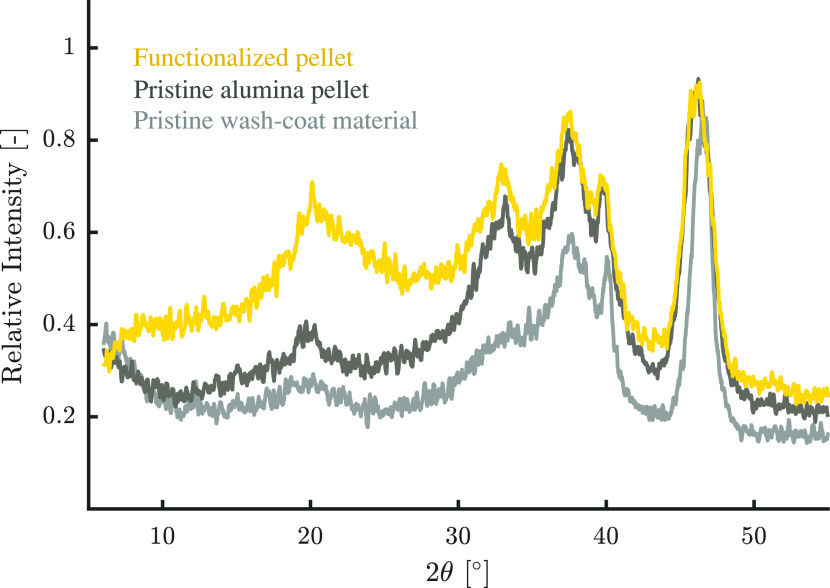
XRD spectra of pristine alumina pellets, wash-coat, and functionalized alumina pellets. A pure, calcined wash-coat material was measured so as to avoid overlapping with the spectrum of mullite.

**Figure 3:**
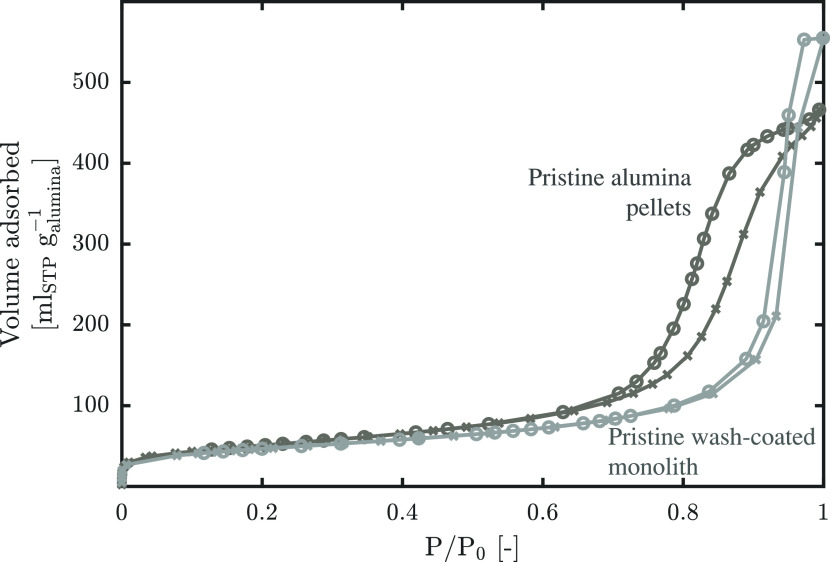
N_2_ isotherms at 77 K of support materials normalized to the mass of alumina.

Alumina was chosen as a support material in this work due to the aforementioned favorable porous properties together with higher hydrothermal stability compared to several widespread silicas. To confirm the suitability of the support material and the presence of the γ phase, X-ray diffraction (XRD) spectra and N_2_ sorption isotherms at 77 K were measured on both the pellets and the wash-coated monolith. For the XRD measurement, a sample of pure, calcined wash-coat material (not deposited on a mullite monolith) was used so as to avoid any overlapping of spectra, while the N_2_ sorption was performed on a wash-coated monolith. The XRD measurements presented in Figure 2 correspond well to those reported in the literature for γ-alumina.^21,63^ The N_2_ sorption isotherms displayed in Figure 3 further confirm the suitability of the material as a support material. Here, the wash-coated monolith was used for the measurement and the adsorbed volume was normalized to the mass of the wash-coat material to enable a more intuitive comparison with the pellets. The shapes of the isotherms are both IUPAC type IV(a) with type H1 hysteresis, indicating a mesoporous material with a narrow range of pore sizes and minimal pore network effects.^64^ Typical for these materials is high surface area and large pore volume, which are deemed ideal for further functionalization.^24^ The values obtained from the analysis of the isotherms are reported in Table 1 along with those of the functionalized samples. The values are again normalized to the mass of alumina for intuitive comparison.

**Table 1:**
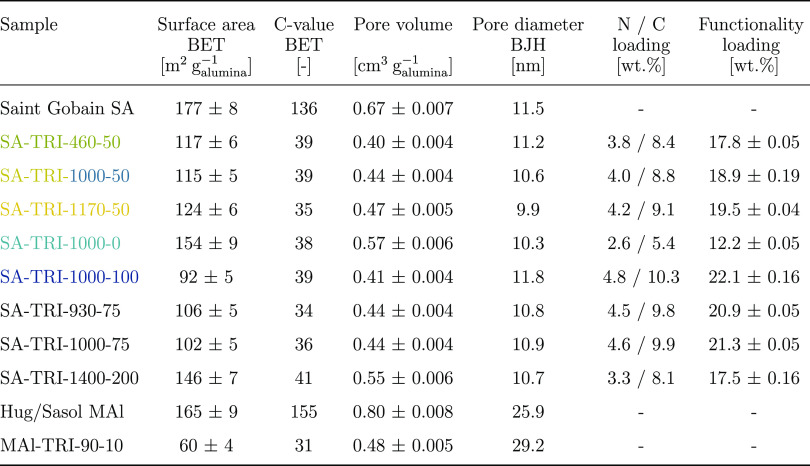
Porous Structure and Elemental Analysis of Pristine and Functionalized Materials^a^

a The colors correspond to those used in the N_2_ sorption isotherms in Figure 4, where applicable.

Both support materials exhibit similarly high surface area and pore volume, although it should be noted that they are not as high as some of those used as a support for other amine-functionalized pellets.^15^ In the case of the pellets, this may be a consequence of pelletization and possible use of non-porous binder materials used in producing the pellets.^51^ For the wash-coat material, this is mostly a consequence of the large pores. These larger pores will likely enhance mass transfer and possibly lead to a higher uptake.^65^ In terms of functionalization, the slightly larger pore volume of the monolith is also expected to enhance the functionalization. An interesting point to note is the very similar C-values of the two support materials. This can indicate similar binding energies of the adsorbate to the surface.^64^ Their value also indicates that the surface area can be approximated with relatively high accuracy using the BET assumption, as the transition from monolayer to multilayer adsorption is well distinguishable.^64^

### Functionalized Pellets

In the open literature, sorbents for CO_2_ capture have generally been functionalized and studied in the form of a powder and, in some cases, formed into pellet materials thereafter.^51,52^ In this work, the materials are functionalized after being formed into the final geometry as this allows for calcining at higher temperatures, thus increasing the mechanical stability of the sorbent. To investigate whether this method has an adverse effect on the adsorption properties, some pellets were crushed to a powder and functionalized in the powder form using otherwise identical conditions as for pellets. The CO_2_ adsorption capacities at 0.37 mmol g_sorbent_^–1^ and 22 Pa CO_2_ partial pressure were within 0.4% of each other, showing that this has little to no adverse effect on the CO_2_ adsorption capacity.

The CO_2_ adsorption capacity of the functionalized pellets was then compared to that of the pristine pellets. The latter was found to be 0.07 mmol g_sorbent_^–1^ compared to the 0.47 mmol g_sorbent_^–1^ of the functionalized pellets at 42 Pa, i.e., only 13% of the capacity. Functionalization with an octyl chain (OTMS) containing no amine moieties further reduced the CO_2_ adsorption capacity, namely to 0.005 mmol g_sorbent_^–1^, i.e., 1% of the amine-functionalized pellets, thus confirming the enhancement of CO_2_ adsorption by the amine moieties.

#### N_2_ Isotherms

**Figure 4:**
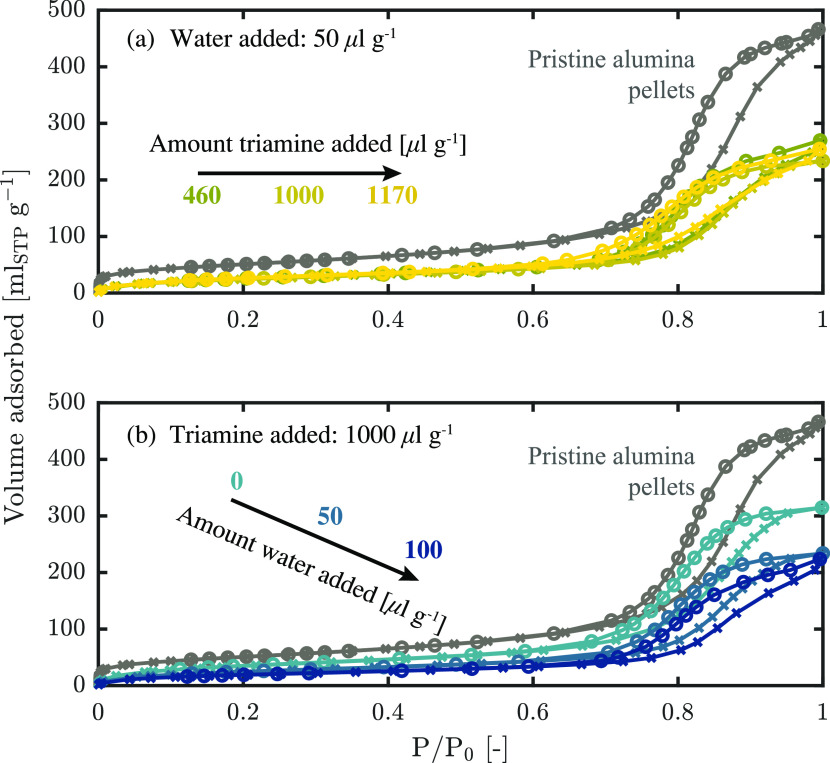
N_2_ isotherms at 77 K of pellets functionalized with (a) different amounts of amine and a constant amount of 50 μL g_alumina_^–1^ water added and (b) different amounts of water and a constant amount of 1000 μL g_alumina_^–1^ of amine added.

N_2_ sorption isotherms at 77 K of various functionalized pellets, shown in Figure 4, are similar in shape to those of the support material, indicating that the homogeneous cylindrical pore structure does not change upon functionalization. This observation is underlined by the XRD measurements, displayed in Figure 2, which show that the crystalline structure of the support is maintained after functionalization.

Figure 4a shows the isotherms of pellets functionalized with different amine concentrations. Interestingly, varying the triamine concentration by a factor of 2.5 does not significantly change the isotherm. A small change can be discerned at the highest triamine concentration, where a small shift of the hysteresis loop to lower pressures indicates a slight broadening of the pore size distribution to smaller pores. Figure 4b shows that varying the water concentration from 0 to 100 μL g_alumina_^–1^ has a more significant effect. Most noticeable is the downward shift of the isotherms with increasing water concentration. This reflects a decrease in both surface area and pore volume. A slight shift of the hysteresis loop at the highest water concentration to higher pressures can also be discerned, indicating a shift of the pore size distribution to larger pore sizes.

The quantitative analysis of the isotherms is shown in Table 1. The results are normalized to the mass of alumina by subtracting the mass of the functional groups, which is calculated using results obtained from elemental analysis. This isolates the effect of functionalization on specific surface area and pore volume from the increase in material density. The elemental analysis results of nitrogen and carbon were compared and revealed that the grafting was likely 25% tris- and 75% bis-grafting. This information was then used to adjust the molar mass of the functional group to calculate the mass percentage on the sorbent. The results are reported in Table 1 and in the Supporting Information in Figures S4–S6.

As seen from the isotherms, varying the amine concentration does not have a significant effect on surface area and pore volume, although an increase of amine concentration does lead to a slight increase in amine loading.

In contrast, an increase in water added per gram alumina is shown to significantly decrease both the surface area and pore volume and significantly increase amine loading. This is in line with similar observations in the literature.^30^ This can indicate both the preferential pathway of condensation via hydrolysis and the presence of multilayer reactions shown in Figure 1, both of which require water as a catalyst (condensation) or reagent (multilayer reaction). However, further increasing the water concentration is shown to decrease the amine loading. This can be explained by an increase in polymerization outside of the support material, leading to the formation of the precipitates by polymerization shown in Figure S8. Elemental analysis of these white precipitates confirmed that they are indeed polymerized amines.

Interestingly, the C-value obtained from the BET analysis changes significantly upon functionalization, irrespective of the functional group used. The high C-values obtained for pristine alumina indicate a fairly well-defined transition from monolayer to multilayer adsorption and the presence of similar high-energy adsorption sites.^64^ C-values in the low range observed for functionalized samples indicate a significant overlap of monolayer and multilayer adsorption, making the estimation of the surface area less accurate.^64,66^ The overlap of adsorption mechanisms indicates the presence of adsorption sites of significantly different energies, such as the NH*_x_* and alumina OH groups.

The pore diameters calculated using the BJH method exhibit an expected decrease upon functionalization. However, an increase in pore diameter with increasing loading does not correspond to the assumption of ideal grafting in which even layers of molecules cover the support surface. Rather, this could be a consequence of the filling of smaller pores by local multilayer reactions. This would increase the average pore diameter that subsequently does not include the smaller blocked pores, while still explaining the reduction of the specific surface area and pore volume. The pore size distribution curves shown in Figure S3 in the Supporting Information further support this assumption.

#### DRIFT Measurements

**Figure 5:**
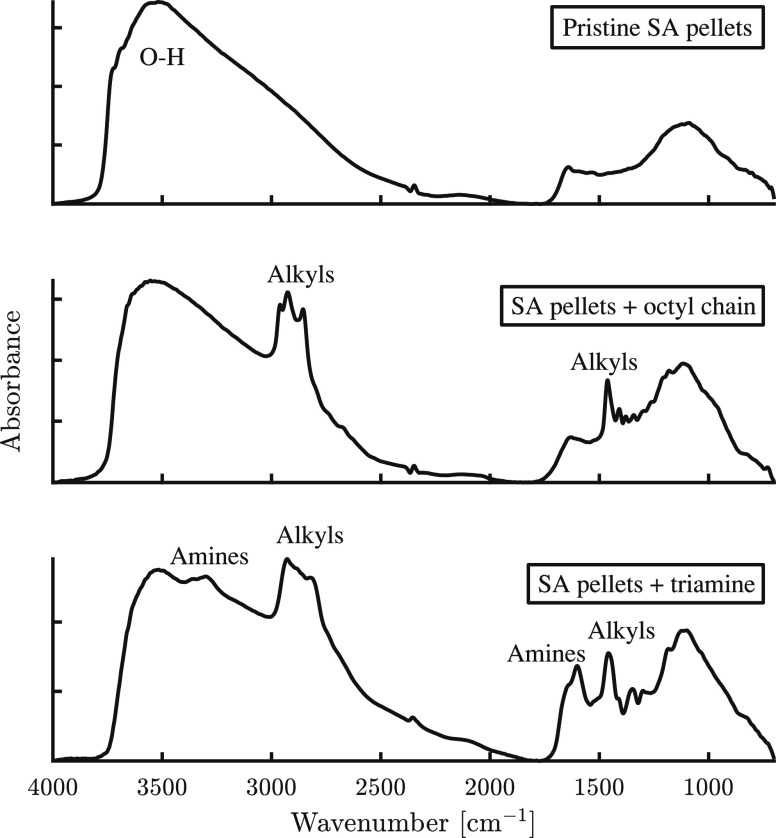
DRIFT spectra of pristine and functionalized pellets, one with an octyl chain (SA-OTMS-1000-75) and one with triamine (SA-TRI-930-75).

DRIFT spectra of pristine and functionalized pellets are shown in Figure 5, and the assignment of absorption bands is summarized in Table S1. The spectra are similar to those seen in the literature for amine-functionalized oxides.^34,51,67−72^ Typical of oxide materials is the broad peak between 3700 and 3450 cm^–1^, indicating the presence of the surface OH groups.^73−75^ The bands between 3000 and 2800 cm^–1^ are attributed to the alkyl chains of the functional groups. Interestingly, the band at 2962 cm^–1^, attributed to CH_3_ stretching,^73^ is missing from the SA-TRI samples. This can indicate that most methoxy groups were consumed by the grafting process. The prominent peak at 2925 cm^–1^ is attributed to the CH_2_ stretching.^51,73,76^ The absorption bands around 3360 and 3301 cm^–1^ are the stretching frequencies of NH and associated NH_2_,^67,73,76^ which are not present in the pristine and octyl-functionalized samples.

At the lower end of the spectra, the prominent peak around 1460 cm^–1^ is likely CH_2_ deformation.^73,76,77^ The absorption bands at 1640 and 1602 cm^–1^ are attributed to NH*_x_* deformation,^51,67,73,76^ which is consistent with their absence from pristine and octyl-functionalized samples. The broad peak between ca. 1150 and 1050 cm^–1^ can be attributed to interacting OH deformation, although possibly also overlapping with Si–O–CH_3_ and Si–O–Si frequencies.^73^ Overall, the spectra show that the pellets were successfully functionalized.

#### CO_2_ Adsorption

**Figure 6:**
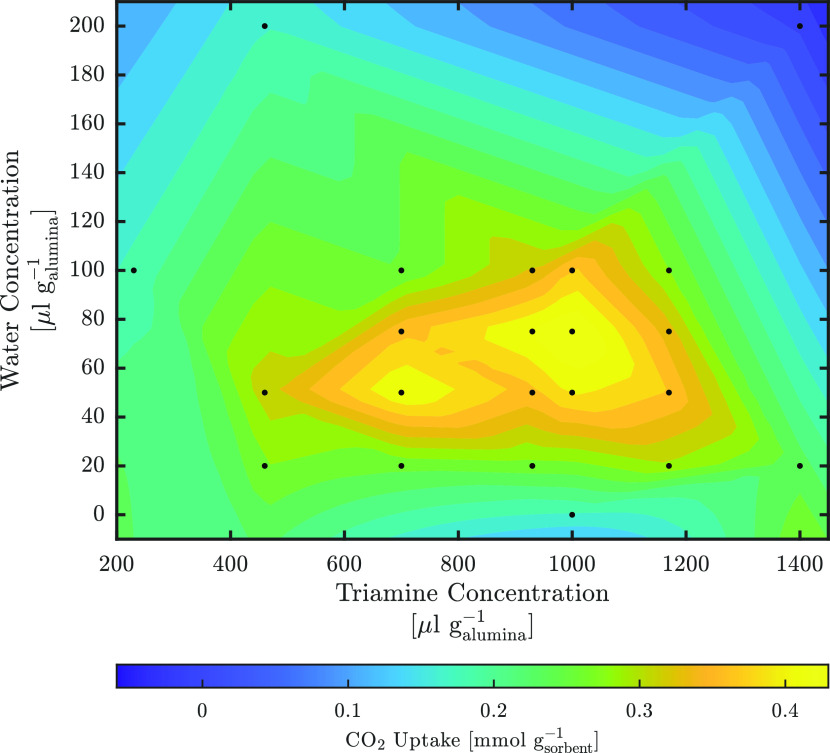
Dry CO_2_ adsorption capacity at 400 ppm sorbents prepared with different amounts of triamine and water. The surface is interpolated between the measurement points, indicated as black dots.

To assess the effect of reagent concentrations on the grafting, both water and triamine concentrations were varied in the grafting of the pellets and the CO_2_ adsorption capacity at 40 Pa was used as an initial performance indicator. The results of 22 experiments are shown in Figure 6. Consistent with the previous observations,^30^ the CO_2_ capacity at 40 Pa exhibits a higher sensitivity to the water concentration during grafting than the triamine concentration. With one exception, 75 μL g_alumina_^–1^ water yields the highest CO_2_ capacities irrespective of the amount of triamine added. Except at very low (<460 μL g_alumina_^–1^) values, the CO_2_ capacity is only slightly affected by the triamine concentration. The maximum CO_2_ capacity was found at 0.46 mmol g_sorbent_^–1^ for the sample SA-TRI-1000-75. The concentration of water used in this sample corresponds to 1.5 monolayers of water adsorbed onto the support material prior to amine addition rather than to the 1.15 monolayers found to be optimal for triamine on MCM-41 silica by Harlick and Sayari.^30^ This discrepancy is likely due to the difference in the support material, as alumina can have significantly different surface basicity compared to silica.^63^

The efficiency of CO_2_ adsorption on the sorbent was then calculated from the nitrogen elemental analysis results using the following relationship, defined as the amine efficiency
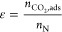
1This number indicates how efficiently the amines are used for CO_2_ capture. A maximum efficiency of 16.3% was calculated for sample SA-TRI-700-50, the sample with the third highest CO_2_ capacity at 40 Pa. A summary of the amine efficiencies of all the samples can be found in Table S7 in the Supporting Information. The results are in line with literature values of similar materials.^33,63^

The concentrations of 930 μL g_alumina_^–1^ triamine and 75 μL g_alumina_^–1^ water were chosen for upscaling experiments, as they offered the best combination of high uptake at 40 Pa and fast adsorption kinetics (discussed in a later section). Roughly 187 g of sorbent was produced in a 7 L reactor using the techniques described in the Methods section. The amount of reagents was kept constant with respect to the mass of alumina. As water is very insoluble in toluene, it is likely mostly present on the alumina surface. Both volumetric and standard dynamic breakthrough measurement techniques were used to quantify the CO_2_ capacity of the resulting sorbent, giving a capacity of 0.46 mmol g_sorbent_^–1^ and 0.44 mmol g_sorbent_^–1^, respectively. This aligns well with the capacity achieved in the small-scale experiments. The slight increase for the upscaled experiment could be due to higher control over process conditions such as efficient mixing by a stirrer, more controlled addition of reactants using a syringe pump, and the use of an argon atmosphere. The difference in capacities obtained from the two different measurement techniques may be attributed to breakthrough capacity being evaluated at *C*_out_/*C*_in_ = 0.95.

Table 2 shows a comparison of the adsorbent produced and characterized in this work compared to other DAC adsorbents in the pellet form presented in the literature. The CO_2_ capacity of the adsorbents produced in this work is in line with most of those with similar oxide support materials and amine functional groups. An exception is the PE-MCM-41 functionalized with TRI, which exhibits an exceptionally high CO_2_ capacity, likely due to the more than 4 times higher pore volume of the support material. However, these pellets are much smaller in comparison, and it has been observed by others that pelletization of grafted powders can lead to lower uptakes and slower kinetics, most likely due to the blockage of pores.^51,52^ Furthermore, the addition of binder materials to increase the mechanical stability of the pellets will further decrease the specific CO_2_ capacity.

**Table 2:** Literature Comparison of Pellet-Shaped, Amine-Functionalized Adsorbents for DAC^a^

			dry CO_2_ capacity at 25 °C			
support	amine	pellet size [mm]	400 ppm [ mmol g_sorbent_^–1^]	pore volume [cm^3^ g^–1^]	surface area BET [m^2^ g^–1^]	ref
MCF silica	PEI	1.8	1.0	0.02 (3.21)	3.1 (594)	(17)
NFC	AEAPDMS	10	0.94		7.1 (26.8)	(16)
silica gel	diamine	2–5	0.4	0.65 (1.07)	216 (422)	(78)
PE-MCM-41	TRI	0.25–0.4	0.98	0.87 (3.09)	367 (1230)	(15)
SBA-15	APTES		0.09			(79)
fumed silica	PEI	>1.7	1.67	0.4	27.2	(19)
γ-alumina	PEI	0.15–0.8	1.23	0.04 (0.69)	4.7 (244)	(18)
SBA-15	APS	0.15–0.5	0.53	0.40 (1.04)	232 (860)	(80)
γ-alumina	TRI	3	0.46	0.35 (0.67)	84 (177)	this work

a Values in brackets are those of the corresponding pristine support material.

The highest capacities are achieved by sorbents with PEI as a functional group. These extraordinarily high capacities are generally achieved over very long adsorption times, as the mass transfer kinetics have been observed to be very slow in these sorbents.^19^ This is likely due to pore blockage by the amines, seen by the extremely low surface area of such sorbents, forcing the CO_2_ to diffuse through the aminopolymers and creating this increased mass transfer resistance.

A parameter that is rarely mentioned in the academic literature is the mechanical stability of the sorbents. This is an important aspect for longevity and use in the industry. The crush strength of the pellets was given as 12.3 lb (5.6 kg). The pellet sorbents presented in this work can therefore be assumed to be mechanically stable and of a size that allows for low pressure drops, while maintaining a CO_2_ capacity comparable to similar sorbents in the open literature.

#### Nature of CO_2_ Adsorption

**Figure 7:**
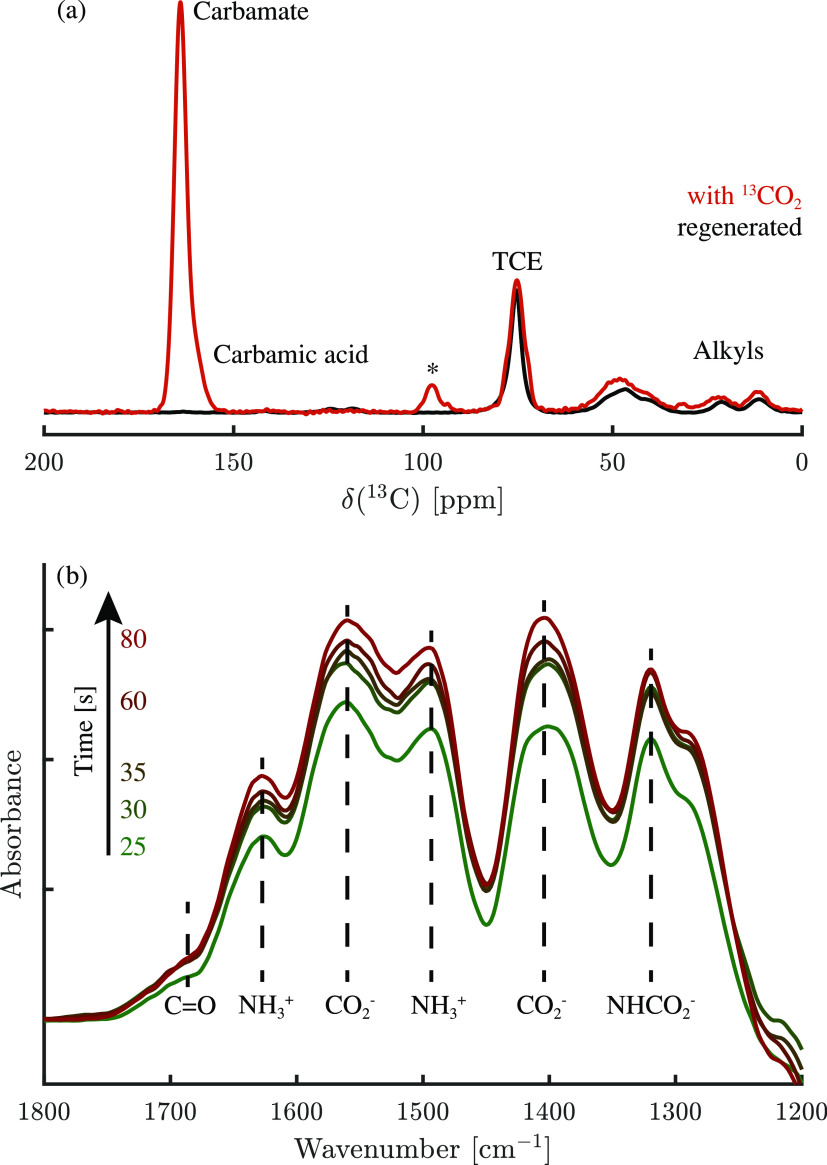
Spectroscopic analysis of CO_2_ adsorption: (a) ^13^C DNP-enhanced NMR spectra of crushed SA-TRI-1000-75 pellets (black) and SA-TRI-1000-75 pellets + adsorbed ^13^CO_2_ (red) and (b) time-resolved DRIFT spectra of SA-TRI-1000-75 pellets during CO_2_ adsorption at 400 ppm; the spectra are shown without scaling after subtracting from the original spectra the regenerated SA-TRI-1000-75 pellet spectrum obtained at *t* = 0.

To understand the mechanisms of adsorption on the grafted materials, dynamic nuclear polarization surface-enhanced NMR spectroscopy (DNP-SENS) was used. This methodology has shown to be indispensable for addressing the structure of surface species, thanks to the selective enhancement of the NMR signal coming from surface sites.^58−61^ As the focus of our studies was alkylamine chains, we resorted to ^13^C and ^15^N DNP-enhanced NMR spectroscopy. For the materials studied, DNP enhancements were found to be in the range of 9–22 (measured for the ^1^H signal of TCE), which helped the observation of surface species with high spectral quality in the case of ^13^C and enabled the observation of the signal for ^15^N, which would be impossible otherwise. The measurements were performed on both regenerated and ^13^CO_2_-adsorbed samples. The ^13^CO_2_ adsorption was performed as close to dry DAC conditions as possible on crushed pellets, with an equilibrium pressure of 100 Pa reached during adsorption. The results are shown in Figure 7a, and an overview of the resonances observed in the literature is reported in the Supporting Information, Table S3. The chemical shifts of the regenerated sample show the CH_2_–NH as a shoulder around 50 ppm, the CH_2_–NH_2_ at 42 ppm, and alkyl groups at 21 and 11 ppm of the grafted triamine in both regenerated and loaded samples.^33,81^ The peak at 47 ppm possibly indicates the presence of some unreacted methoxy groups.^82^ The resonances appearing at 164 ppm for the sample with adsorbed ^13^CO_2_ are attributed to carbamate.^83,84^ A slight shoulder at around 160 ppm is attributed to the presence of carbamic acid.^83,84^ The low relative intensity of the carbamic acid peak compared to the carbamate peak is an indication that it is less prevalent and therefore possibly less stable. Interestingly, the shoulder is less intense than that observed by others.^83,84^ As lower pressures were used in this work during adsorption (100 Pa here vs 100 kPa in refs (83, 84)), this could indicate that a higher ratio of carbamate vs carbamic acid can be expected in DAC applications compared to applications at higher CO_2_ concentrations. The prevalence of carbamate was confirmed by further ^15^N DNP-enhanced NMR measurements, which are presented in Figure S9 and show a peak appearing at around 88 ppm corresponding to carbamate formation.^84^ The adsorption was performed under dry conditions; therefore, no bicarbonate formation is expected. However, this cannot be verified due to a significantly attenuated signal using CPMAS NMR and an overlap of resonances with more prevalent species such as carbamate.^85^

FTIR measurements were performed on the same sample used in the NMR measurements, and they confirmed the presence of carbamate while also showing the absence of bicarbonate. The measurements are shown in Figure S10, and the additional measurement of a sample with adsorbed ^12^CO_2_ shows a slight red-shift of the ^13^CO_2_-adsorbed sample. This is expected due to the reduced mass of the ^12^CO_2_.

The results obtained using time-resolved DRIFT measurements during CO_2_ adsorption, shown in Figure 7b, further confirm the results of the NMR measurements. A small shoulder around 1680 cm^–1^ is characteristic of the C=O stretch of carbamic acid,^41,63^ although its relative peak is again small in comparison. The other peaks are assigned to stretch and deformation bands belonging to species present in ammonium carbamate, as indicated in the figure. A summary of these is reported in the Supporting Information, Table S2. The time-resolved measurements show the fast diffusion and reaction rate of the adsorption on the amine-functionalized pellets, as the measurements were not further scaled after baseline correction.

#### Adsorption Kinetics

**Figure 8:**
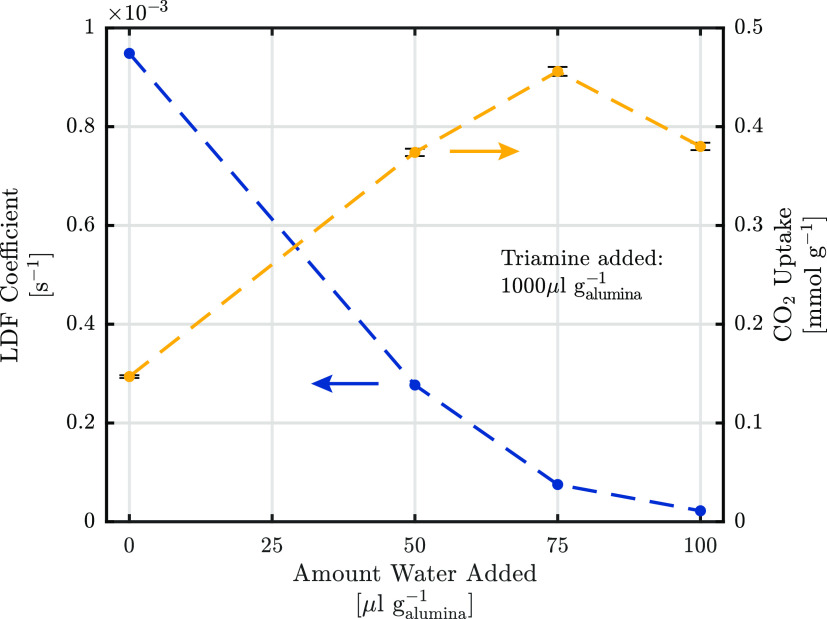
Effect of the amount of water added during preparation on the CO_2_ uptake and mass transfer kinetics. Samples produced with a constant amine addition of 1000 μL g_alumina_^–1^.

A crucial aspect for DAC, which is more difficult to quantify and often overlooked, is the CO_2_ adsorption kinetics.^14^ Here, we investigate the effect of water concentration during grafting on the adsorption kinetics. As water enables multilayer reactions and polymerization of the amines, the water concentration is expected to have a larger effect on the kinetics than the triamine concentration, likewise the results observed as far as the CO_2_ capacity is concerned. The kinetic measurements are performed in batch mode and fitted to the widely used linear driving force (LDF) model assuming changing gas concentration. It should be noted that pure CO_2_ was used for these measurements at a pressure corresponding to CO_2_ partial pressure in air (40 Pa). Consequently, mechanisms involving molecular diffusion, such as film diffusion, are not measured; however, especially for pellets, film diffusion has a minimal impact and is often neglected.^86^ Both macropore and micropore diffusion, which are generally the limiting mass transfer mechanisms in such sorbents, can be measured using this technique as they depend only on the partial pressure of CO_2_. The kinetics were measured on four samples grafted with varying water concentration and equal triamine concentration. The pressure curves are fitted to a LDF batch adsorption model assuming a linear isotherm and isothermal conditions. The model fits the experimental results well, as shown in the Supporting Information, Figure S11, showing that the LDF model provides a good approximation of the mass transfer in these pellets.
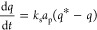
2The kinetic parameter *k*_s_*a*_p_, as defined in eq 2, is estimated from the experimental results and shown in Figure 8. The equilibrium uptake q* is calculated using a linear isotherm model, whereas the uptake *q* is correlated to the measured pressure with a time-dependent mass balance. A significant decrease of the kinetic parameters with increasing water concentration can be observed, which is consistent with further measurements performed but not shown for brevity. This decrease in mass transfer rate is likely due to increased multilayer reactions and polymerization of the triamine within the pores, which likely creates a polymer layer through which the CO_2_ must diffuse to reach the available adsorption sites.^80^ The decrease is also accompanied by an increase in CO_2_ capacity, which can also be explained by increased multilayer reactions and polymerization, indicating a trade-off between the two properties. As the ideal DAC sorbent should exhibit both fast mass transfer rates and high CO_2_ capacity, this trade-off makes the choice of the sorbent grafting nontrivial. The optimal configuration could therefore be indicated by modeling the process.

### Functionalized Monoliths

To assess the similarity in the functionalization procedure between the monolith and the pellets, a monolith was divided into small pieces of ca. 2 mm × 2 mm × 0.4 mm and functionalized with the same concentration of triamine and water per mass of alumina wash coat as the upscaled pellets. The pieces were then characterized using N_2_ sorption, CO_2_ adsorption, and batch kinetic measurements. Similar to the pellets, the functionalized monolith exhibited a decrease in both surface area (−64%) and pore volume (−39%), while the pore diameter estimated with BJH did not change significantly. The CO_2_ capacity at 40 Pa was also comparable to that of the pellets, i.e., of 0.44 mmol g_sorbent_^–1^ when normalized to the mass of the active sorbent (alumina + triamine). The mass transfer coefficient of the monolith estimated by fitting the batch measurements was 1.2 × 10^–3^ s^–1^, i.e., significantly higher than that of the pellets functionalized with the same amounts of water and triamine. This is reasonable as the larger pores in the wash-coat material and the hierarchical structure of the monolith enable faster diffusion. These results indicate that the monolith can be grafted using the same procedure and conditions as the pellets; therefore, no further optimization was performed.

The above-mentioned conditions were then used to graft a larger scale monolith of 30 mm × 30 mm × 130 mm. The functionalization procedure of these was slightly different from that of the pellets as the radially closed structure of the honeycomb monolith meant a dropwise addition of water would not result in an even distribution of water on the wash-coat material within the monolith. Indeed, a low-capacity and uneven CO_2_ uptake along the monolith functionalized using this procedure was measured. To ensure an even water distribution of water on the monolith, a dried monolith was submerged in hot water and then dried to a weight that corresponded to 1.5 monolayers of water. In a separate 160 mm-long, 500 mL reactor, a stirred solution of 500 mL of dried toluene with 16.5 mL of triamine was prepared and heated to 85 °C. The monolith was then placed in the reactor overnight without stirring. This procedure ensured that enough amines were present within all the channels of the monolith without having to diffuse into them, which would cause an uneven distribution as the amines would likely react with the alumina before diffusing to the center of the monolith.

**Figure 9:**
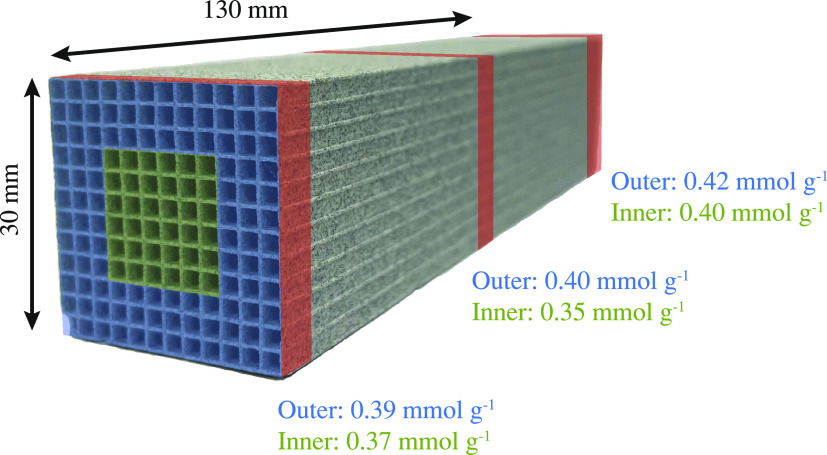
CO_2_ capacity at 40 Pa different sections of the monolith measured using the volumetric technique and normalized to the mass of active sorbent (alumina + triamine). Inner (green) and outer (blue) channels are distinguished.

The evenness of grafting on the monolith was verified quantitatively by measuring the CO_2_ uptake of pieces cut from the monolith at different axial positions and then separated into central and outer channels as illustrated in Figure 9. The final grafting method is shown to give a rather even CO_2_ uptake along the monolith. It can be seen that the central channels still exhibit a slightly lower uptake compared to the outer channels, especially in the central axial position. Aside from leading to a lower uptake, this slight heterogeneity could affect the shape of the breakthrough curve, possibly leading to premature breakthrough of CO_2_. However, as DAC is an extraction of CO_2_ from air with no constraints on the outlet concentration of CO_2_, the monolith can still be saturated further after breakthrough.

**Figure 10:**
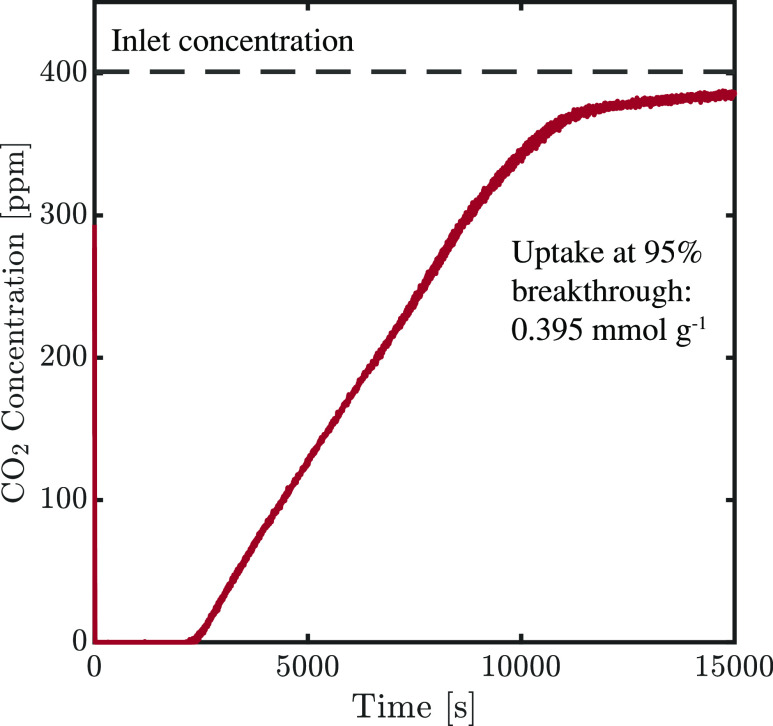
Two breakthrough curves of a monolith at 400 ppm CO_2_ in N_2_ at a flow rate of 0.001 mol s^–1^; the volume of the monolith is 117 cm^3^. The flow is stopped after 4 h 10 min due to very slow diffusion in the last part of the curve.

Before the volumetric measurements, the monolith was characterized using breakthrough measurements to assess the overall CO_2_ uptake and to make a qualitative assessment of the performance under DAC conditions. The breakthrough curve is shown in Figure 10, from which an uptake of 0.4 mmol g_sorbent_^–1^ was calculated, which is similar to that achieved at the 1 g scale and the volumetric measurements. The shape of the breakthrough shows an absence of irregularities, such as those found elsewhere,^49^ and indicates an even distribution of the CO_2_ adsorption and flow along the monolith. Moreover, the long tail after 95% breakthrough seems to indicate that there are two mass transfer regimes, as observed in other supported amine sorbents.^80^ Therefore, the first 95% of the breakthrough is assumed to be dominated by the mass transfer in the gas film and pores, whereas the last 5% is dominated by the mass transfer in the amine layer. These breakthrough experiments did not reach completion, and therefore, the total saturation time is unknown. Nevertheless, it is worth noting that in the design of a cycle, it would not be favorable to load the sorbent to saturation. Furthermore, the uptake at 95% breakthrough corresponds well to the values measured in the volumetric measurements.

### Comparison of Pellets and Monoliths

#### Thermodynamics

**Figure 11:**
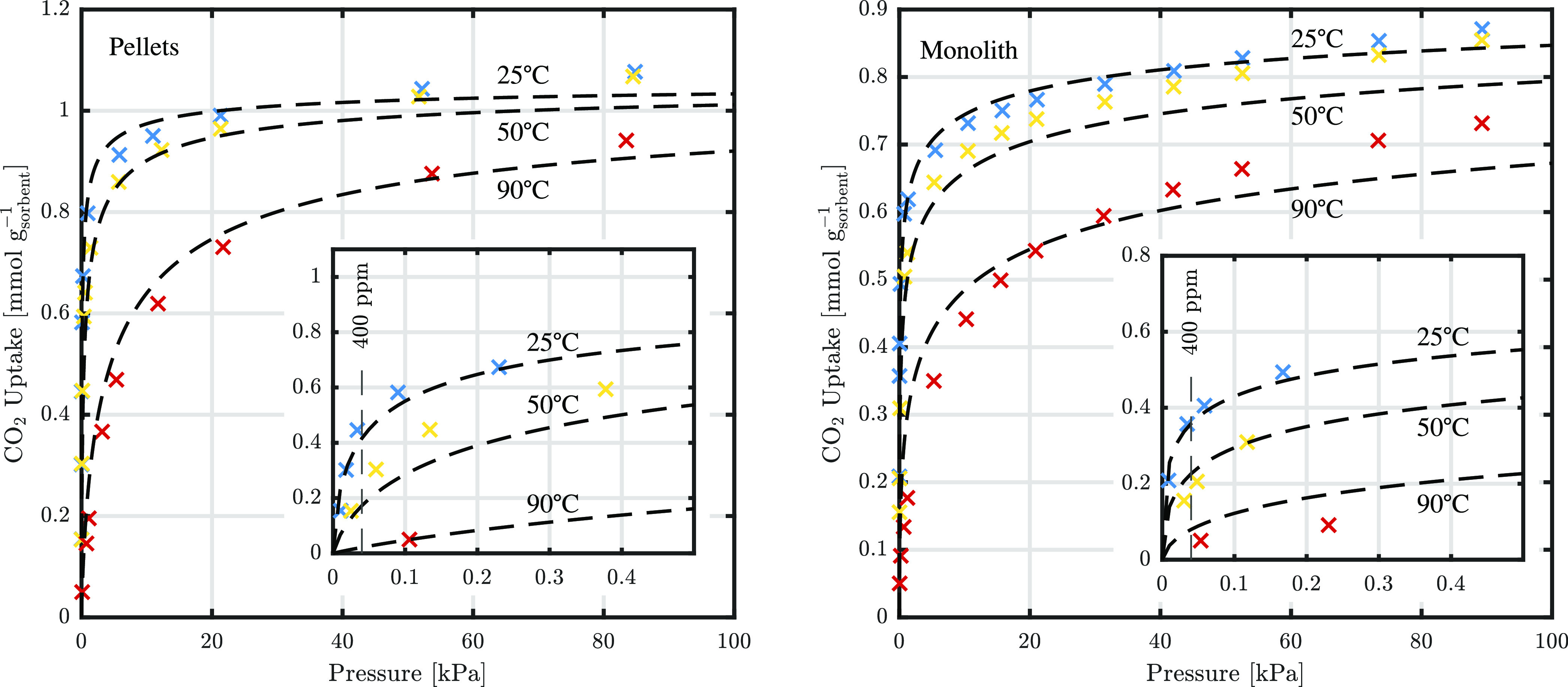
Isotherms of upscaled batch of pellets (left) and whole monolith (right) depicted in Figure 9. A temperature-dependent Toth isotherm was fitted to both sorbents, whose values are shown in Table 3.

The CO_2_ isotherms of both the pellets and the monolith pieces measured at 25, 50, and 90 °C are shown in Figure 11. The favorable isotherm shapes are similar to those seen in the literature for DAC sorbents and demonstrate the feasibility of using the sorbent in a TVSA process.^87^ Furthermore, the uptake of both the pellets and the monolith is very similar at the low partial pressures experienced in DAC, when the uptake is expressed in terms of mass of active sorbent (alumina + triamine). Within the context where different contactor geometries are comparatively assessed, this represents a helpful characteristic. The isosteric heats of adsorption are estimated from these isotherms using the Clausius–Clapeyron equation, giving 70 kJ mol^–1^ for the pellets and 77 kJ mol^–1^ for the monolith. The similar heat of adsorption is to be expected for adsorbents consisting of a similar support material that is functionalized with the same molecule. The slight difference could be due to a different alumina being used, with possibly different surface energies. It should be emphasized that these isotherms are measured under dry conditions. Realistic DAC conditions also include the presence of moisture, which has been seen to increase the capacity of similar materials.^44^ The complex effect of moisture on adsorption is beyond the scope of this study. The measured isotherms were fitted to a temperature-dependent Toth isotherm (eqs 3–6), which is also shown in Figure 11. The corresponding Toth values are reported in Table 3.
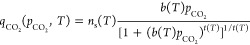
3
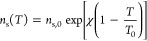
4
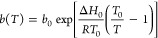
5
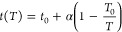
6

**Table 3:** Temperature-Dependent Toth Parameters Fitted for Pellets and the Monolith^a^

parameter	units	value for pellets	value for monolith
*n*_s,0_	mmol g_sorbent_^–1^	1.067	1.025
*b*_0_	kPa^–1^	93.89	6757
*t*_0_		0.461	0.231
*T*_0_	K	298	298
Δ*H*_0_	J mol^–1^	70,000	77,000
χ		6.49 × 10^–13^	4.66 × 10^–10^
α		0.708	0.215
RMSE 25 °C	mmol g_sorbent_^–1^	0.042	0.019
RMSE 50 °C	mmol g_sorbent_^–1^	0.069	0.041
RMSE 90 °C	mmol g_sorbent_^–1^	0.053	0.068

a RMSE refers to the root mean square error.

#### Cyclic Stability

Although a full cyclic stability investigation is beyond the scope of this study, a limited amount of cycles were performed to verify the stability over the amount of cycles performed during characterization. Both breakthrough and volumetric adsorption measurements were repeated over multiple cycles to assess the stability of the sorbent by comparing the CO_2_ uptakes.

Degradation of amine-functionalized sorbents can be due to multiple effects, such as leaching of the amines, oxidation,^88^ or formation of urea.^69,71^ These mechanisms affect the choice of the regeneration procedure, as oxidation has been observed to occur at temperatures above 70 °C, whereas the formation of urea occurs at high CO_2_ concentrations and elevated temperatures but can be suppressed by the presence of moisture.^69,71^ Therefore, care was taken to avoid these conditions during regeneration.

**Figure 12:**
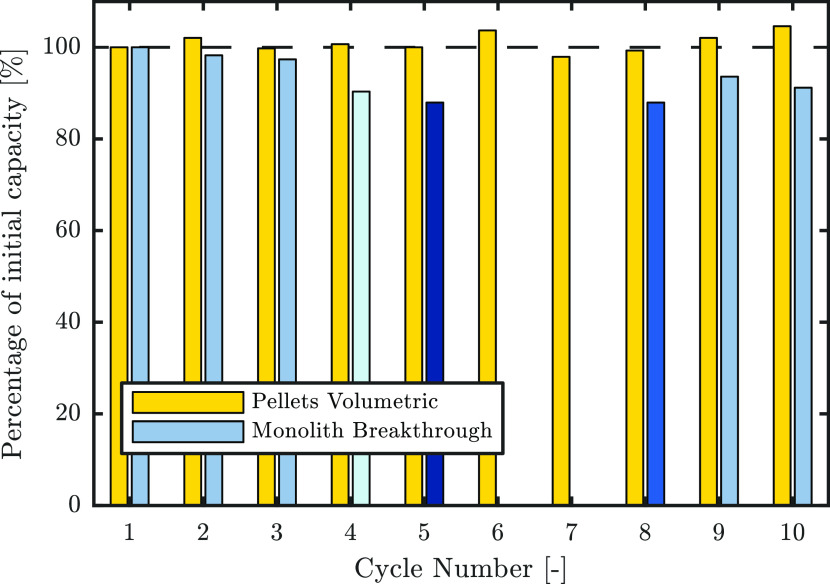
Relative capacity of pellets and of a monolith after 10 repeated cycles using volumetric measurements for the pellets and breakthrough measurements for a monolith at 400 ppm, normalized to the first measurement. The breakthroughs in cycles 6 and 7 were performed using 5.62% CO_2_ in N_2_ and are therefore not comparable. The shading of the monolith breakthrough data indicates the flow rates used: lightest blue = 0.18 mmol s^–1^, light blue = 1.0 mmol s^–1^, dark blue = 2.0 mmol s^–1^, and darkest blue = 3.0 mmol s^–1^.

The results of repeated uptake measurements are reported in Figure 12. Ten cycles were performed using volumetric measurements for the pellets and breakthrough measurements for a monolith. In the case of the breakthroughs, the CO_2_ uptake is calculated at *C*_out_/*C*_in_ = 0.95 at the outlet. The two cycles that are not shown here were performed at a different concentration (5.62% CO_2_ in N_2_) and are therefore not comparable but may still be considered in the cycle count.

The uptakes of the pellets at 40 Pa obtained from the volumetric measurements exhibit little variability in uptake, with a maximum of 4.6% difference. Most significantly, no decrease in uptake is discernible, which would indicate degradation of the sorbent. The breakthrough measurements performed on the monolith exhibit a slightly different picture, as a decrease of 8.8% from the initial CO_2_ uptake can be observed after 10 cycles. The variability in uptake at different flow rates can be explained by the bed being saturated to a different extent at the time defined for the uptake calculation. The slight decrease in capacity could be due to several factors, such as less stable grafting or the breakthrough column not being completely vacuum tight, leading therefore to the presence of oxygen during regeneration at 90 °C causing oxidation.

As the change in uptake in both adsorbents is low, they can be considered stable over the number of cycles necessary for characterization. As mentioned previously, the effect of moisture has not been considered in this work. A stabilizing effect of water has been observed on amine-functionalized sorbents.^44^ Negative effects of species, such as carbonic acid, formed in the presence of moisture cannot be fully excluded, although they are expected to be minor due to the stability of alumina across a broad range of pH.

#### Mass Transfer Kinetics

**Figure 13:**
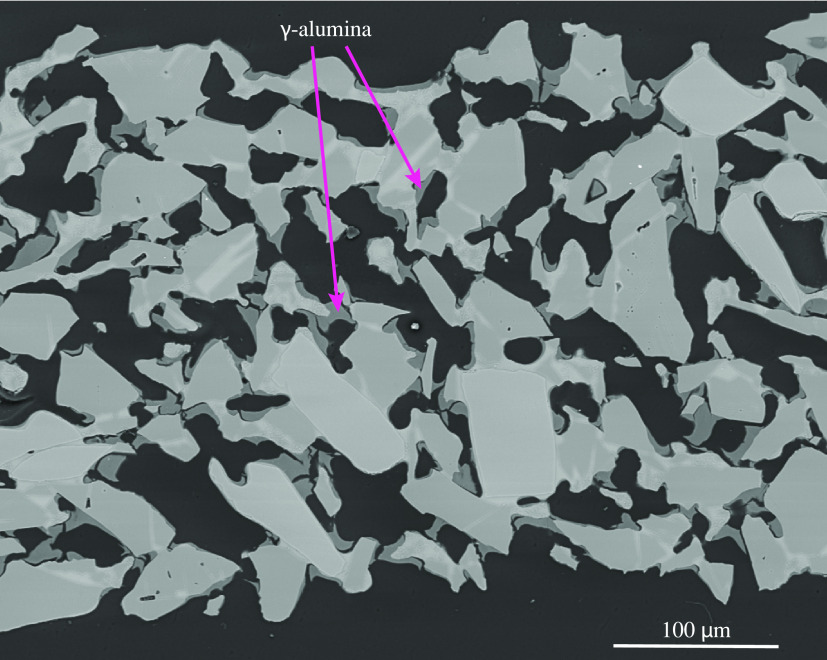
SEM picture of a cross-section of the channel wall of a monolith. The darker patches correspond to the γ-alumina wash-coat integrated in the pores, and the light patches are mullite.

As mentioned previously, kinetics play a crucial role in the DAC process.^14^ They can vary significantly between sorbents but are often difficult to compare quantitatively between different papers in the literature due to the variety of methods used. In this work, the kinetics of two structured sorbents were compared using a batch volumetric uptake measurement as described above. The fitted LDF coefficient *k*_s_*a*_p_ of the pellets was estimated as 1.5 × 10^–4^ s^–1^, whereas the LDF coefficient of the monolith was measured as 1.2 × 10^–3^ s^–1^. This significant increase of kinetics of almost one order of magnitude in the monolith makes them very promising for DAC applications. This difference reflects the difference in nominal diffusion length, which is ca. 1.5 mm in the pellets (Sauter mean radius) compared to ca. 0.2 mm for the monolith (half the wall thickness). Furthermore, SEM pictures of the monolith shown in Figure 13 indicate that the wash-coat material is deposited in small particles of ca. 1 – 2 μm within the macroporous walls of the monolith. The mass transfer resistance in the macropores of the mullite is likely insignificant compared to the diffusion in the mesopores of the alumina, resulting effectively in a much shorter diffusion length of the order of the size of the small particles. With this hypothesis in mind, a pellet was powdered using a pestle and mortar and the same kinetic measurement was performed. Interestingly, the LDF coefficient increased to 1.6 × 10^–3^ s^–1^, which is very similar to that of the monolith. All these results point to macropore diffusion limitations within the pellets, which indicate the importance of the geometry of the contactor for fast mass transfer and therefore efficient DAC. The ability of the monolith to host such small particles without creating a large pressure drop, as would be the case for powders and small pellets, makes it a promising candidate for use in DAC.

A preliminary comparison can be made based on experiments by calculating the productivity of the adsorption step as this includes both thermodynamics and kinetics. The volumetric productivity can be defined as the amount of CO_2_ captured per unit volume of contactor and unit time using the values measured in the breakthrough experiments. The time and amount captured are defined at *C*_out_/*C*_in_ = 0.9, as the kinetics for both materials decline sharply after this. The volume corresponds to the volume of the column occupied by the sorbent. The productivity for the two materials is then 6.48 mol m^–3^ h^–1^ for the pellets and 7.56 mol m^–3^ h^–1^ for the monolith, with a pressure drop of 0.12 bar for the pellets and of 0.013 bar for the monolith. While the volumetric productivity of two contactors is similar, the energy penalty is much lower with the monolith due to the lower pressure drop, further underlining its promising characteristics for use in DAC.

## Conclusions

The motivation for this study stems from the need to address the challenges associated with direct air capture processes, which are related to the high volumetric flow rates involved and the slow kinetics of adsorption. To overcome these obstacles, improved contactors are necessary. Monoliths have been identified as a promising option for DAC applications due to their low pressure drop and established use in catalytic conversion in the industry. In this study and in the context of DAC, we have demonstrated a method for grafting amines onto alumina-coated monoliths post-extrusion, which allows for the use of industrial materials as a support, avoids calcination of the amines, and achieves homogeneous functionalization across the entire structure.

To this end, this work has aimed to demonstrate the successful amine-grafting process on commercial alumina pellets, which was thoroughly characterized to determine the sorbent properties. The CO_2_ uptake capacity was found to be most sensitive to the amount of water added in the grafting procedure, with the highest uptake found at 1.5 monolayers of water. The techniques have been successfully used and adapted to graft amines onto alumina-coated honeycomb monoliths. The CO_2_ amounts adsorbed per unit mass of active sorbent were similar between pellets and monoliths and could be described using the Toth isotherm. Moreover, such an amount adsorbed was shown to be quite homogeneous within the monolith, which have exhibited significantly enhanced adsorption kinetics compared to the pellets, as observed both in batch and breakthrough experiments.

In conclusion, the study has shown that amine-grafted alumina-coated monoliths are promising for utilization as sorbents and contactors for CO_2_ capture from air. To assess their viability in real DAC industrial processes, their long-term stability is important, whose characterization was beyond the scope of this study. One key limitation of this study includes the absence of moisture in cyclic stability measurements and its effect on adsorption thermodynamics and mass transfer kinetics. Nonetheless, prior work in the literature indicates that the presence of moisture can have a positive effect on these aspects. Therefore, we believe that these findings represent a significant step in the use of monoliths for DAC and open the door toward the optimization of the monolith structure and the identification of the most promising cycle and process design for their implementation in direct air capture contactor applications.

## Supporting Information Available

Additional information on methods and materials, functionalized pellets, spectroscopy measurements, and mass transfer kinetics (PDF)
