# Supplementary material for: Developing Versatile Contactors for Direct Air Capture of CO2 through Amine Grafting onto Alumina Pellets and Alumina Wash-Coated Monoliths
Source: Ind Eng Chem Res. 2023 Aug 15;62(34):13594–611. doi: 10.1021/acs.iecr.3c01265 (PMC10472440; doi:10.1021/acs.iecr.3c01265)
Supplement: Supplementary file 1 — ie3c01265_si_001.pdf [file ie3c01265_si_001.pdf]

Supplementary Information: Developing Versatile Contactors for Direct Air Capture of CO_2 through Amine Grafting onto Alumina Pellets and Alumina Wash-Coated Monoliths

Quirin Grossmann,† Valentina Stampi-Bombelli,† Alexander Yakimov,‡ Scott Docherty,‡ Christophe Copéret,‡ and Marco Mazzotti*,†

†*Institute of Energy and Process Engineering, ETH Zurich, 8092 Zurich, Switzerland*

‡*Department of Chemistry and Applied Biosciences, ETH Zurich, 8093 Zurich, Switzerland*

E-mail: marco.mazzotti@ipe.mavt.ethz.ch

Methods & Materials

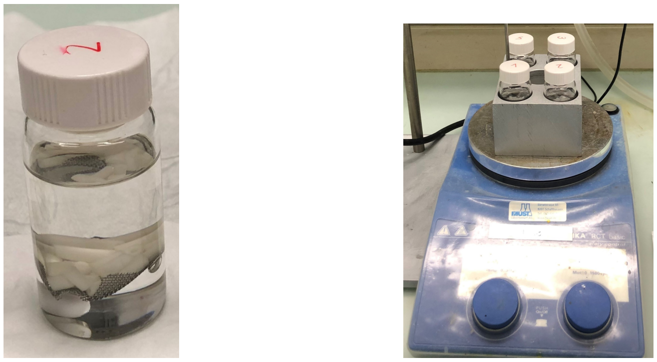

Figure S1: Left: vial with pellets separated from stir bar by a mesh. Right: temperature-controlled reaction block with 4 vials.

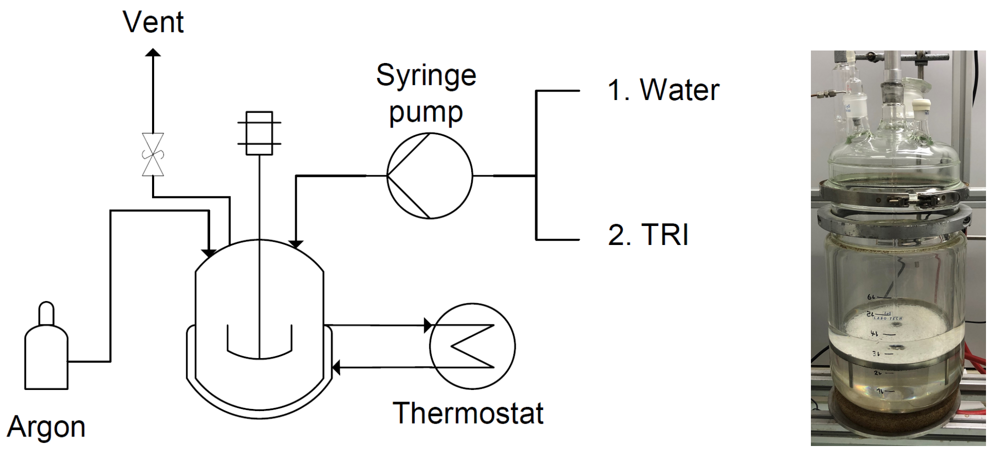

Figure S2: Left: schematic of bench-scale functionalization setup. Right: 7 l reactor with pellets.

Functionalized Pellets

Pore Size Distribution

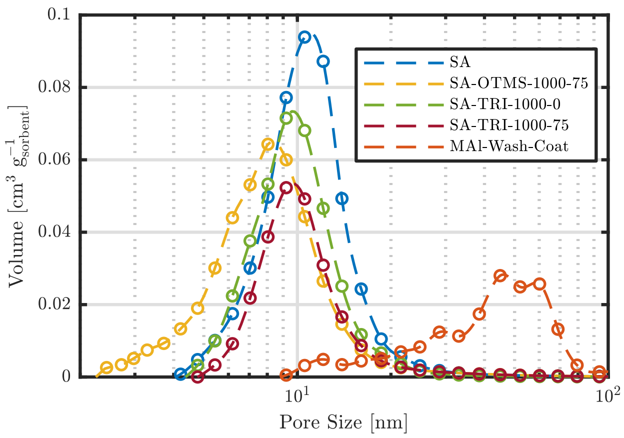

Figure S3: BJH pore size distribution of selected sorbents

Elemental Analysis

Elemental analysis results of nitrogen and carbon were then used to calculate the amount of functional groups grafted onto the pellets. By determining the number of mols of triamine both nitrogen and carbon masses correspond to, it could be estimated how many methoxy groups were consumed. This analysis revealed that the grafting was likely 25% tris- and

H_2O [μl g^{-1}_{al}] \ TRI [μl g^{-1}_{al}]	230	460	700	930	1000	1170	1400
0					2.6%		
20		3.4%	3.2%	3.1%		3.3%	3.3%
50		3.8%	3.7%	4.2%	4.0%	4.2%	
75			4.4%	4.5%	4.6%	4.3%	
100	2.3%		3.7%	3.9%	4.8%	3.8%	
200		3.9%					3.8%

Figure S4: Nitrogen mass percent of functionalized pellets determined by elemental analysis.

H_2O [μl g^{-1}_{al}] \ TRI [μl g^{-1}_{al}]	230	460	700	930	1000	1170	1400
0					5.4%		
20		7.4%	7.3%	7.2%		7.6%	7.1%
50		8.4%	8.9%	9.2%	8.8%	9.1%	
75			10.2%	9.8%	9.9%	9.3%	
100	4.8%		8.5%	8.5%	10.3%	8.7%	
200		8.3%					8.1%

Figure S5: Carbon mass percent of functionalized pellets determined by elemental analysis.

H_2O [μl g^{-1}_{al}] \ TRI [μl g^{-1}_{al}]	230	460	700	930	1000	1170	1400
0					12.2%		
20		15.7%	14.7%	14.5%		15.5%	15.2%
50		17.8%	17.4%	19.4%	18.8%	19.5%	
75			20.6%	20.9%	21.2%	20.0%	
100	10.8%		17.1%	18.3%	22.2%	17.8%	
200		18.4%					17.5%

Figure S6: Aminosilane mass percent of functionalized pellets calculated assumine 25% tris-grafting and 75% bis-grafting.

H_2O [µl g^{-1}_{al}] \ TRI [µl g^{-1}_{al}]	230	460	700	930	1000	1170	1400
0					9.8%		
20		11.7%	11.8%	12.3%		12.8%	10.5%
50		11.9%	16.3%	12.5%	14.2%	12.7%	
75			11.9%	13.5%	14.2%	11.3%	
100	11.7%		9.9%	11.8%	11.4%	10.4%	
200		6.6%					0.4%

Figure S7: Amine efficiency of CO_2 adsorption on all samples.

75% bis-grafting. This result was used to calculate the mass added through functionalization. Similar to the CO_2 adsorption results, they show a high sensitivity of the aminosilane content on the water addition. Interestingly, in the case of 75 μl $g^{-1}_{alumina}$ water added, the number of mols of water added is approximately equal to three times the amount of TRI molecules grafted, *i.e.* equal to the number of methoxy groups in the grafted TRI molecules. This could indicate that the amount of amine added should be in reference to the amount of water added.

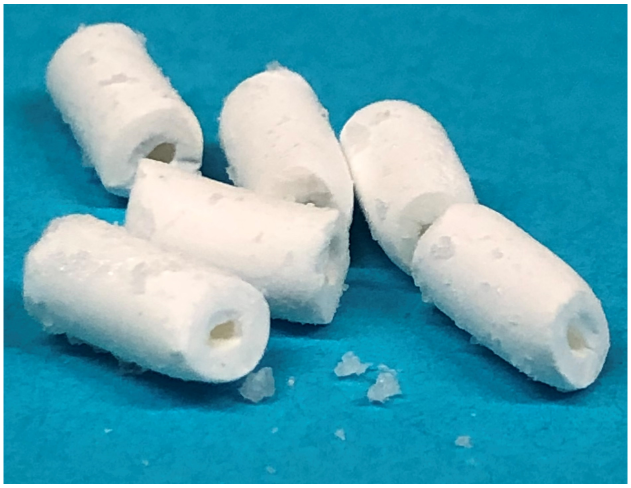

Figure S8: Pellets grafted with an excessive amount of water (200 μl $g^{-1}_{alumina}$), showing polymerization on the outside of the rings.

Spectroscopy

Table S1: FTIR/DRIFT absorption band assignment on CO_2-free samples.

Frequency [cm^{-1}]	Observed in	Assignment
3724 & 3681	SA	Free Al-OH species at 3714[1]
3700 - 3450	SA, SA-TRI, SA-OTMS	Stretching modes of interacting OH groups[2–4]
3360 & 3301	SA-TRI	NH & associated NH_2 stretching[3,5–7]
2962	SA-OTMS	CH_3 stretching[3]
2925	SA-TRI, SA-OTMS	CH_2 stretching[3,5,7,8]
2856	SA-OTMS	CH_2 stretching[3,7]
2820	SA-TRI	CH_2 stretching[5,7,8]
1640	SA-TRI	NH deformation[3,5,6]
1602	SA-TRI	NH_2 deformation[3–10]
1460	SA-TRI, SA-OTMS	CH_2 & OCH_3 deformation[3–5,7,8,11]
1150 - 1050	SA, SA-TRI, SA-OTMS	Interacting OH deformation[3], Si-O-C stretching & C-O stretching[5]

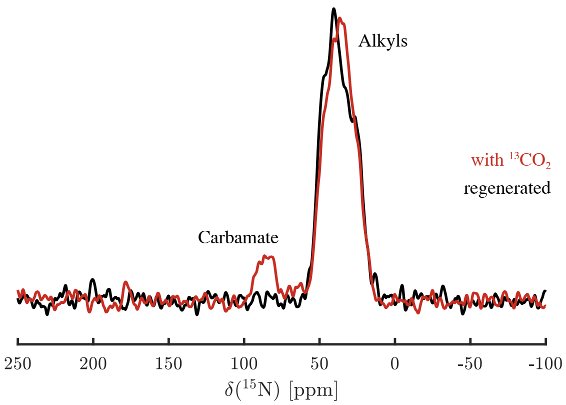

Figure S9: ^{15}N DNP-enhanced NMR spectra of crushed SA-TRI-1000-75 (black) and SA-TRI-1000-75 with adsorbed $^{13}CO_2$ (red)

Table S2: FTIR/DRIFT absorption band assignment on CO_2 -adsorbed samples.

Frequency [cm^{-1}]	Species	Material	Assignment
1715-1600(1710)	Bound carbamate	APS on alumina	C=O stretch[12,13]
1700-1680	Carbamic acid	APS on alumina	C=O stretch[4,12,14]
1660-1630	Carbamate	-	NH_3^+ [6,7,15]
1650	Bicarbonate	APS on alumina	Carboxylate on surface hydroxyl groups[16]
1635-1625	NH_3^+	APS on alumina	NH_3^+ deformation[4,6,12,17]
1570-1545	Carbamate	APS on alumina	COO^- [4,6,12,14,17]
1550-1485	NH_3^+	APS on alumina	NH_3^+ asym. deformation[4,6,12,17,18]
1535-1510	Bound carbamate	APS on alumina	NH def./C-N stretch[12,14,16]
1488	Carbamate	-	COO^- sym. stretch[19]
1480	Carbamate	-	$NHCOO^-$ deformation[18]
1440	Bicarbonate	APS on alumina	Carboxylate on surface hydroxyl groups[16]
1430	Carbamate	APS on alumina	COO^- sym. stretch[4,6,12,14,17]
1420-1335	Carbamate	APS on SBA-15	COO^- sym. strech[20]
1380	Carbamate	APS on alumina	COO^- sym. stretch[4,6,12,16,17] or $NCOO^-$ skeletal vibration[12,19]
1320	Carbamate	APS on alumina	$NCOO^-$ skeletal vibration[12,15,21] or CN stretch[12]

Table S3: NMR shift assignment on $^{13}CO_2$ -adsorbed samples.

Shift [ppm]	NMR Type	Material	Assignment
9.7, 11.5	^{13}C	APTES on clay, APS on MCF	$-CH_2-Si$[22,23]
21.4, 21.8 – 23.4	^{13}C	APTES on clay, APS on MCF	–CH–[22,23]
13,23	^{13}C	APS on alumina	CH_2 groups[10,16]
40 - 44	^{13}C	-	Alkyl groups adjacent to primary amines[16,22,24,25]
50 - 53	^{13}C	MAPS on MCF	Alkyl groups adjacent to secondary amines[23,24]
**160, 161**	^{13}C	APS on SBA15/clay	Carbamic acid[26,27]
163	^{13}C	APS on alumina	"Chemisorbed CO_2"[16]
**163.8**	^{13}C	APS on alumina	Bicarbonate[27]
**164.2**	^{13}C	APS on alumina	Carbamate[26,27]
**164.6**	^{13}C	APS on MCM-41	Carbamate[10]
165	^{13}C	APS on silica	Carbamate[8,10,28]
24 - 44	^{15}N	-	Alkylamines[27]
88	^{15}N	-	Ammonium carbamate[27]

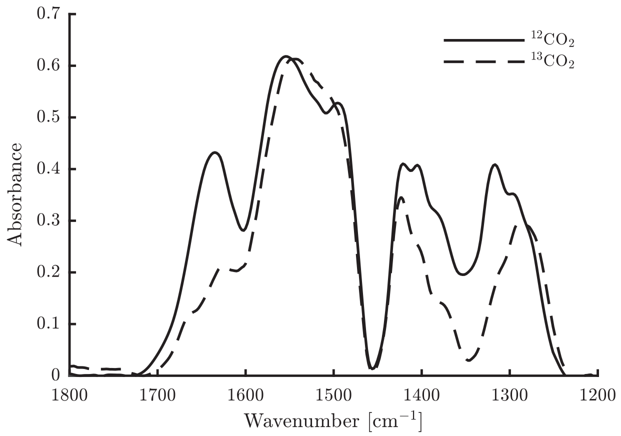

Figure S10: FTIR spectra of crushed SA-TRI-1000-75 with adsorbed $^{12}CO_2$ (solid) and $^{13}CO_2$ (dashed). The spectra are shown after subtracting the spectrum of regenerated SA-TRI-1000-75.

Mass Transfer Kinetics

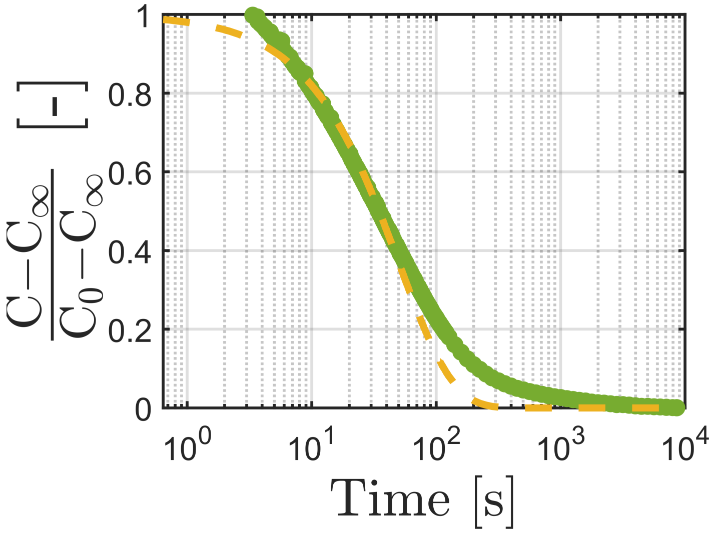

Figure S11: Fitted batch LDF model in yellow (–) and measurement points in green.

References

(1) Khaleel, A. A.; Klabunde, K. J. Characterization of Aerogel Prepared High-Surface-Area Alumina: In Situ FTIR Study of Dehydroxylation and Pyridine Adsorption. *Chem. Eur. J.* **2002**, *8*, 3991–3998.

(2) de Melo Pinheiro, A. F.; Nijmeijer, A.; Sripathi, V. G. P.; Winnubst, L. Chemical modification/grafting of mesoporous alumina with polydimethylsiloxane (PDMS). *Eur. J. Chem.* **2015**, *6*, 287–295.

(3) Bellamy, L. *The infra-red spectra of complex molecules*; Chapman and Hall: London, 1975; Vol. 1; pp 13–34, 277–291.

(4) Knöfel, C.; Martin, C.; Hornebecq, V.; Llewellyn, P. L. Study of carbon dioxide adsorption on mesoporous aminopropylsilane-functionalized silica and titania combining microcalorimetry and in situ infrared spectroscopy. *J. Phys. Chem. C* **2009**, *113*, 21726–21734.

(5) Chiang, C.-H.; Ishida, H.; Koenig, J. L. The structure of γ-aminopropyltriethoxysilane on glass surfaces. *J. Colloid Interface Sci.* **1980**, *74*, 396–404.

(6) Hiyoshi, N.; Yogo, K.; Yashima, T. Adsorption characteristics of carbon dioxide on organically functionalized SBA-15. *Microporous Mesoporous Mater.* **2005**, *84*, 357–365.

(7) Sayari, A.; Heydari-Gorji, A.; Yang, Y. CO_2induced degradation of amine-containing adsorbents: reaction products and pathways. *J. Am. Chem. Soc.* **2012**, *134*, 13834–13842.

(8) Drage, T. C.; Arenillas, A.; Smith, K. M.; Snape, C. E. Thermal stability of polyethylenimine based carbon dioxide adsorbents and its influence on selection of regeneration strategies. *Microporous Mesoporous Mater.* **2008**, *116*, 504–512.

(9) Yu, Q.; Delgado, J. d. l. P.; Veneman, R.; Brilman, D. W. Stability of a benzyl amine based CO_2 capture adsorbent in view of regeneration strategies. *Ind. Eng. Chem. Res.* **2017**, *56*, 3259–3269.

(10) Sayari, A.; Belmabkhout, Y. Stabilization of amine-containing CO_2 adsorbents: dramatic effect of water vapor. *J. Am. Chem. Soc.* **2010**, *132*, 6312–6314.

(11) Bellamy, L.; Williams, R. Infrared spectra and polar effects. Part III. Internal spectral relationships. *J. Chem. Soc. (Resumed)* **1956**, 2753–2757.

(12) Potter, M. E.; Cho, K. M.; Lee, J. J.; Jones, C. W. Role of Alumina Basicity in CO_2 Uptake in 3-Aminopropylsilyl-Grafted Alumina Adsorbents. *ChemSusChem* **2017**, *10*, 2192–2201.

(13) Hedin, N.; Bacsik, Z. Perspectives on the adsorption of CO_2 on amine-modified silica studied by infrared spectroscopy. *Curr. Opin. Green Sustain. Chem.* **2019**, *16*, 13–19.

(14) Bacsik, Z.; Ahlsten, N.; Ziadi, A.; Zhao, G.; Garcia-Bennett, A. E.; Martín-Matute, B.; Hedin, N. Mechanisms and kinetics for sorption of CO_2 on bicontinuous mesoporous silica modified with n-propylamine. *Langmuir* **2011**, *27*, 11118–11128.

(15) Wang, X.; Schwartz, V.; Clark, J. C.; Ma, X.; Overbury, S. H.; Xu, X.; Song, C. Infrared study of CO_2 sorption over "molecular basket" sorbent consisting of polyethylenimine-modified mesoporous molecular sieve. *J. Phys. Chem. C* **2009**, *113*, 7260–7268.

(16) Bali, S.; Leisen, J.; Foo, G. S.; Sievers, C.; Jones, C. W. Aminosilanes grafted to basic alumina as CO_2 adsorbents—role of grafting conditions on CO_2 adsorption properties. *ChemSusChem* **2014**, *7*, 3145–3156.

(17) Bacsik, Z.; Atluri, R.; Garcia-Bennett, A. E.; Hedin, N. Temperature-induced uptake of CO_2 and formation of carbamates in mesocaged silica modified with n-propylamines. *Langmuir* **2010**, *26*, 10013–10024.

(18) Tumuluri, U.; Isenberg, M.; Tan, C.-S.; Chuang, S. S. In situ infrared study of the effect of amine density on the nature of adsorbed CO_2 on amine-functionalized solid sorbents. *Langmuir* **2014**, *30*, 7405–7413.

(19) Yu, J.; Chuang, S. S. The structure of adsorbed species on immobilized amines in CO_2 capture: an in situ IR study. *Energy Fuels* **2016**, *30*, 7579–7587.

(20) Danon, A.; Stair, P. C.; Weitz, E. FTIR study of CO_2 adsorption on amine-grafted SBA-15: elucidation of adsorbed species. *J. Phys. Chem. C* **2011**, *115*, 11540–11549.

(21) Didas, S. A.; Sakwa-Novak, M. A.; Foo, G. S.; Sievers, C.; Jones, C. W. Effect of amine surface coverage on the co-adsorption of CO_2 and water: spectral deconvolution of adsorbed species. *J. Phys. Chem. Lett.* **2014**, *5*, 4194–4200.

(22) Pinto, M. L.; Pires, J.; Rocha, J. Porous materials prepared from clays for the upgrade of landfill gas. *J. Phys. Chem. C* **2008**, *112*, 14394–14402.

(23) Didas, S. A.; Kulkarni, A. R.; Sholl, D. S.; Jones, C. W. Role of amine structure on carbon dioxide adsorption from ultradilute gas streams such as ambient air. *ChemSusChem* **2012**, *5*, 2058–2064.

(24) Badertscher, M.; Bühlmann, P.; Pretsch, E. *Structure Determination of Organic Compounds: Tables of Spectral Data*; Springer: Berlin, 2009; Vol. 1; p 117.

(25) Ek, S.; Iiskola, E. I.; Niinistö, L.; Vaittinen, J.; Pakkanen, T. T.; Root, A. A 29Si and 13C CP/MAS NMR study on the surface species of gas-phase-deposited γ-aminopropylalkoxysilanes on heat-treated silica. *J. Phys. Chem. B* **2004**, *108*, 11454–11463.

(26) Pinto, M. L.; Mafra, L.; Guil, J. M.; Pires, J.; Rocha, J. Adsorption and activation of CO_2 by amine-modified nanoporous materials studied by solid-state NMR and $^{13}CO_2$ adsorption. *Chem. Mater.* **2011**, *23*, 1387–1395.

(27) Chen, C.-H.; Shimon, D.; Lee, J. J.; Didas, S. A.; Mehta, A. K.; Sievers, C.; Jones, C. W.; Hayes, S. E. Spectroscopic characterization of adsorbed $^{13}CO_2$ on 3-aminopropylsilyl-modified SBA15 mesoporous silica. *Environ. Sci. Technol.* **2017**, *51*, 6553–6559.

(28) Chen, C.-H.; Shimon, D.; Lee, J. J.; Mentink-Vigier, F.; Hung, I.; Sievers, C.; Jones, C. W.; Hayes, S. E. The "missing" bicarbonate in CO_2 chemisorption reactions on solid amine sorbents. *J. Am. Chem. Soc.* **2018**, *140*, 8648–8651.
